# Epidemiological overview of major depressive disorder in Scandinavia using nationwide registers

**DOI:** 10.1016/j.lanepe.2023.100621

**Published:** 2023-03-28

**Authors:** Joëlle A. Pasman, Joeri J. Meijsen, Marit Haram, Kaarina Kowalec, Arvid Harder, Ying Xiong, Thuy-Dung Nguyen, Andreas Jangmo, John R. Shorter, Jacob Bergstedt, Urmi Das, Richard Zetterberg, Ashley Tate, Paul Lichtenstein, Henrik Larsson, Ingvild Odsbu, Thomas Werge, Ted Reichborn-Kjennerud, Ole A. Andreassen, Patrick F. Sullivan, Alfonso Buil, Martin Tesli, Yi Lu

**Affiliations:** a Department of Medical Epidemiology and Biostatistics, Karolinska Institutet, Sweden; b Institute of Biological Psychiatry, Mental Health Center Sct. Hans, Mental Health Services Copenhagen, Roskilde, Denmark; c The Lundbeck Foundation Initiative for Integrative Psychiatric Research (iPSYCH), Copenhagen, Denmark; d Department of Mental Disorders, Norwegian Institute of Public Health, Oslo, Norway; e Division of Mental Health and Addiction, Oslo University Hospital, Norway; f College of Pharmacy, University of Manitoba, Canada; g Department of Science and Environment, Roskilde University, Denmark; h Unit of Integrative Epidemiology, Institute of Environmental Medicine, Karolinska Institutet, Stockholm, Sweden; i School of Medical Sciences, Örebro University, Örebro, Sweden; j Lundbeck Foundation GeoGenetics Centre, GLOBE Institute, University of Copenhagen, Copenhagen, Denmark; k Department of Clinical Medicine, Faculty of Health and Medical Sciences, University of Copenhagen, Copenhagen, Denmark; l NORMENT Centre, Institute of Clinical Medicine, University of Oslo and Division of Mental Health and Addiction, Oslo University Hospital, Norway; m K.G. Jebsen Centre for Neurodevelopmental Disorders, University of Oslo and Oslo University Hospital, Oslo, Norway; n Departments of Genetics and Psychiatry, University of North Carolina at Chapel Hill, NC, USA; o Centre of Research and Education in Forensic Psychiatry, Oslo University Hospital, Oslo, Norway

**Keywords:** Major depressive disorder, Electronic health records, National patient register, Epidemiology, Heritability

## Abstract

**Background:**

Major depressive disorder (MDD) is a common psychiatric disorder associated with a high disease burden. This study gives a comprehensive overview of the prevalence, outcomes, treatment, and genetic epidemiology of MDD within and across the Scandinavian countries.

**Methods:**

This study has aimed to assess and compare across Norway, Denmark, and Sweden 1) the prevalence and trajectories of MDD and comorbidity, 2) outcomes and treatment, and 3) heritability (Denmark and Sweden only). The analyses leveraged data on 272,944 MDD cases (and 6.2 million non-cases) from Norway, Sweden, and Denmark in specialist care in national longitudinal health registers covering 1975–2013. Relying on harmonized public data global comparisons of socioeconomic and health metrics were performed to assess to what extent findings are generalizable.

**Findings:**

MDD ranked among the most prevalent psychiatric disorders. For many cases, the disorder trajectory was severe, with varying proportions experiencing recurrence, developing comorbid disorders, requiring inpatient treatment, or dying of suicide. Important country differences in specialist care prevalence and treatment were observed. Heritability estimates were moderate (35–48%). In terms of socioeconomic and health indices, the Scandinavian nations were comparable to one another and grouped with other Western nations.

**Interpretation:**

The Scandinavian countries were similar with regards to MDD epidemiological measures, but we show that differences in health care organization need to be taken into consideration when comparing countries. This study demonstrates the utility of using comprehensive population-wide registry data, outlining possibilities for other applications. The findings will be of use to policy makers for developing better prevention and intervention strategies.

**Funding:**

10.13039/501100004359Swedish Research Council (Vetenskapsrådet, award D0886501 to PFS), 10.13039/100000002US National Institutes of Mental HealthR01 MH123724 (to PFS), European Union’s Horizon 2020 Research and Innovation Program (847776 and 964874, to OA) and 10.13039/501100000781European Research Council grant (grant agreement ID 101042183, to YL).

**Research in context**

**Evidence before this study**

Major depressive disorder (MDD) is a highly prevalent and disabling disorder, persistent across nations, cultures, and time. The understanding of epidemiological profiles and heritability is important to inform clinical guidelines and policy makers, and for future analyses of genetic architecture. The uniform health care system and available registry data in the Scandinavian countries provide a unique possibility to identify a wide set of epidemiological outcomes in MDD such as prevalence, comorbidity, clinical outcomes, treatment trajectories and heritability, and to present cross-country comparisons. We searched MEDLINE, Embase and APA PsychInfo with no date or language restrictions (see Table S0a, Fig. S0 for the full search strategy) for reviews and original studies mapping nationwide epidemiological outcomes in MDD with the use of registries or health records. The search retrieved 359 unique articles. Among the 324 original articles, 78 studies investigated epidemiological outcomes in MDD and included nationwide cohort samples (Table S0b). As expected, the main countries providing nationwide data were Sweden and Denmark with 64 studies, and 1 Norwegian study was identified. Beyond the Scandinavian countries, a total of 13 studies used nationwide samples from Finland, Iceland, Netherlands, Austria and South Korea. Among the 78 original research articles investigating MDD outcomes, 18 studies investigated >1 outcome, but none provided a complete overview of prevalence, comorbidity, clinical outcomes, treatment trajectories and heritability of MDD. Moreover, no study compared MDD outcomes with other nations.

**Added value of this study**

With comprehensive nationwide registry data, we aligned definitions of study cohorts and statistical methods between the three Scandinavian countries to provide cross-country comparisons of a broad set of epidemiological outcomes in MDD. We integrated global comparisons of socioeconomic status, mortality and morbidity to demonstrate the comparability of the Scandinavian countries with other high-income countries. Combined, we found that the prevalence of MDD is moderate in Scandinavia with a substantial sex-imbalance. Based on the longitudinal registries, we found high rates of severe clinical and socioeconomic outcomes among individuals with experience of MDD. The high rate and pattern of comorbid mental disorders was similar across countries, and heritability estimates of 35–48% aligned with estimates found in other cohorts. We describe important differences in the national health services between countries that could explain differences found in prevalence of MDD and other mental disorders as well as clinical outcomes.

**Implications of all the available evidence**

Given the complex and heterogeneous nature of MDD, the understanding of epidemiological outcomes and genetic architecture rely on large sample sizes. Our comprehensive epidemiological overview of MDD in Scandinavia add value to this understanding and show the possibilities and limitations of nationwide registry-based research. The cross-country comparisons performed highlight that the knowledge of health care services and paths to care are of great importance to the evaluation of outcomes and should be considered in future epidemiological research of MDD.

## Introduction

The Scandinavian countries Norway, Sweden, and Denmark share a common history going back thousands of years.^1^ Due to their common ancestry, the Scandinavian population shares a genetic background and has remained partly genetically distinct from other European populations.^2^^,^^3^ The Scandinavian countries bear strong similarities in government, culture, and economics. Common cultural characteristics include, for example, individualism, high trust in the government, and gender egalitarianism.^4^^,^^5^ The languages show resemblance to such an extent that speakers can often understand each other. Norway, Sweden, and Denmark share consistent positions among the top 10 of various world-rankings on happiness and well-being^6^ and they are among the wealthiest in the world.^7^

Healthcare is similarly organized across Scandinavia. It is funded by the government via taxation and allows near-universal access to medical care. Health care expenditures currently constitute around 10.1–11.4% of the gross domestic product in Norway, Denmark, and Sweden.^8^^,^^9^ The health care system in Scandinavia is organized into primary and specialist care. Basic psychiatric health care is provided in primary care, while specialist psychiatric care comprises inpatient hospitals and outpatient clinics and requires a referral paper from a general practitioner. Data on health care usage, redeemed prescriptions, and population demographics (e.g., birth, death, education) are collected in numerous detailed registries.^10^

Major depressive disorder (MDD) is characterized by episodes of depressive mood and/or loss of interest and pleasure. The ability to concentrate, psychomotor activity, sleep and eating patterns can be impaired, and unreasonable feelings of guilt, low self-esteem and suicidal thoughts may be present (International Statistical Classification of Diseases and Related Health Problems [ICD]^11^). In 2019, the global age-standardized one-year prevalence was estimated at 3.4% (Global Burden of Disease [GBD] 2019^12^). Depressive disorders accounted for the largest proportion (37%) of psychiatric disorder-related disability-adjusted life years.^12^ Outcomes for those with MDD vary, with some developing life-long maladies with significant functional impairment, whereas others experience a single, mild episode that spontaneously remits.

Given the burden that MDD places on individual lives and society, it is paramount to increase insight into the presentation, prevalence, and etiology of the disorder.^13^ For genetic studies, it is generally assumed that Scandinavian populations are similar to each other and to other western countries, but outside of the reports from governmental agencies, few studies have systematically compared population characteristics of MDD within and across Scandinavian countries (see ‘Research in Context’). Understanding differences and similarities in what an MDD diagnosis constitutes in different countries is important to judge the generalizability of research findings.

The aims of the current study were three-fold: 1. Provide an extensive overview of the prevalence and comorbidity of MDD and compare them across the Scandinavian countries; 2. Assess the association of MDD with treatment and clinical and socioeconomic outcomes; and 3. Estimate to what extent genetic liability contributes to MDD in Scandinavia. As an ancillary aim, we evaluate how the Scandinavian countries compare to the rest of the world in terms of socioeconomic status and mortality/morbidity, to assess to what extent results may be generalizable. Understanding the occurrence, course, and consequences of MDD is pivotal in informing clinical practice and policy.

## Methods

A detailed overview of the included analyses and data sources is given in Table S1 and the Supplementary Notes (numbered Note S1–S7 below).

### Global comparisons of socioeconomic indicators and health

As a first step, we compared the Scandinavian countries to each other and the rest of the world in terms of socioeconomic status, mortality, and morbidity using harmonized data from the GBD study^12^ and the Our World in Data organization.^14^ These efforts have brought together data from numerous governmental and scientific sources and harmonized them to enable country comparisons. To evaluate socioeconomic status, we used eleven key metrics with complete data in 2019 in 168 countries from Our World in Data (Table S2). For a few measures where 2019 data were missing, we brought forward data from the nearest non-missing year for that country. We reduced data complexity to two dimensions using Uniform Manifold Approximation and Projection (UMAP, https://cran.r-project.org/web/packages/umap/vignettes/umap.html^15^) and investigated how countries clustered together on these dimensions with Hierarchical Density-Based Spatial Clustering of Applications with Noise (HDBSCAN; Note S1) using the standardized key metrics.

Global data on mortality, morbidity, and prevalence of MDD in 204 countries were retrieved from the 2019 GBD study (Note S1, Table S3). UMAP and HDBSCAN were again used to cluster countries in terms of their population's health (Note S1). For MDD as well as the most common other psychiatric disorders we extracted estimates of one-year prevalence in Scandinavia (Note S1).

### Study population: data from the national registers

**Fig. 1:**
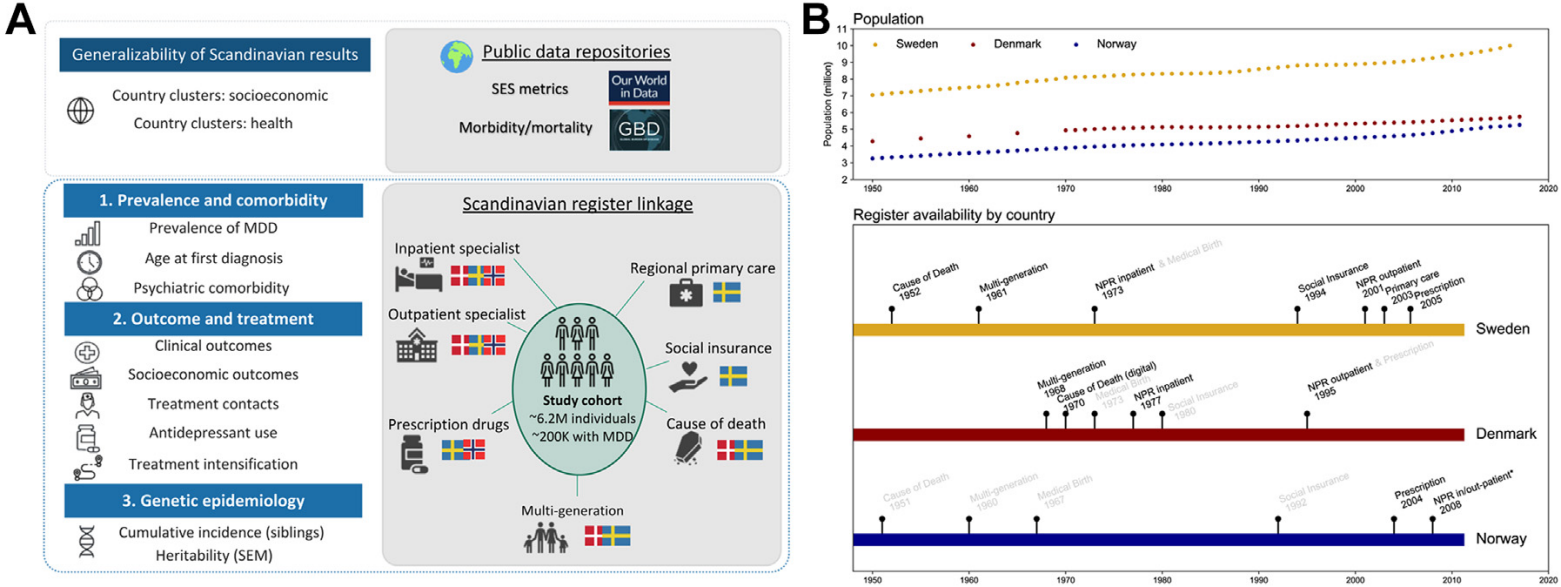
Aims and data sources. **Panel A)** Data sources per aim (1–3), with country flags indicating data availability in each country (blue-yellow = Sweden; red-white = Denmark; red-white-blue = Norway). **Panel B)** Overview of population size (top panel) and register data availability from 1950 onwards in the Scandinavian countries (bottom panel; in grey the registers that were not used for this study). ∗Register contains information regarding cause of death down to 1875 and was computerized as of 1970. ∗∗Available for MoBa sub sample, refer to Note S3.

To capture data on diagnostic information, outcomes, treatment, and genetic epidemiology we used the respective national health registries.^10^ An overview of the registers is given in Fig. 1 and more information can be found in Note S2 and Table S4.

We selected a birth cohort of 1965–2000 for most analyses in MDD cases, limiting ourselves to the decades for which we had appropriate register coverage. In several analyses we excluded cases with less than 5 years follow-up data after their first MDD diagnosis due to death, emigration, or end of register coverage. Note that for the analysis focusing on clinical outcomes we did not exclude individuals who died of suicide, given that this was an outcome of interest. Again, to ensure sufficient data coverage, we excluded individuals who were not born in the respective country in the Danish and Swedish data. Given that MDD diagnostic codes translate poorly to individuals below age 10, we excluded individuals that received a diagnosis before age 10 in most analyses. Following these criteria, we selected a cohort of MDD cases aged 10–49. The Norwegian prevalence and comorbidity analyses relied on registry data linked to the ‘Norwegian Mother, Father, and Child Cohort Study’ (MoBa,^16^ Table S4, Note S3). A detailed overview of cohort selection criteria used in the different analyses can be found in Table S1.

### Aim 1. Prevalence of MDD and comorbidity in specialist care

To assess the prevalence of MDD in the specialist care registries, we extracted individuals with MDD and their age at first diagnosis in the period covered by the registers using ICD-10: F32/F33 codes (or equivalent in ICD-9 and ICD-8; Table S5) with subcodes that clinical features of MDD. We describe the raw prevalence, age distribution and sex differences across the countries (Note S4a–d). To put the prevalence of MDD in perspective, we extracted the prevalence of other common psychiatric disorders from ICD-10 (Table S5). For this overview we focused on individuals born after 1985, after which the ICD-10 coverage became similar between the countries (Table S1). We reported the number of diagnoses in 2013 of MDD, autism spectrum disorder (ASD), attention-deficit/hyperactivity disorder (ADHD), anxiety disorder, bipolar disorder, eating disorder, schizophrenia/schizoaffective disorder, and substance use disorder stratified by sex and age. Finally, we estimated the cumulative incidence of MDD occurring after onset of another disorder, and, vice versa, the cumulative incidence of another disorder after MDD diagnosis (Note S4e–g).

### Aim 2. Outcomes and treatment

We used registry data from Denmark and Sweden to investigate the occurrence of binary clinical outcomes within MDD cases, focusing on recurrence, diagnostic cross-over, self-harm, suicide, and mortality (Note S5a–b). We tested country differences in rates of clinical outcomes while controlling for age and sex. Using Swedish data, we furthermore contrasted socioeconomic outcomes for MDD cases versus non-cases, focusing on benefit payments, sickness leave, income, and educational attainment (Note S5c–d). We tested the impact of MDD on sickness leave, income, and educational attainment using linear regression models and its impact on benefit payments using logistic regressions while controlling for birth year and sex in all models.

We assessed specialist treatment use in Denmark and Sweden, and primary care treatment in Stockholm County, Sweden (Note S6a). The Swedish and Norwegian (aggregated) prescription drug registers were used to present the prevalence of prescription drug use for MDD (Note S6b–c). Furthermore, we reported on common treatment intensification trajectories in Sweden (Note S6d).

### Aim 3. Genetic epidemiology (Denmark and Sweden)

Using genealogical information^17^ and specialist care diagnoses we estimated the heritability of MDD in Denmark and Sweden. For these analyses we compared phenotypic similarity (MDD status) with genetic similarity (comparing the general population with full-siblings, 50% genetically similar, and maternal half-siblings, 25% genetically similar; see Table S6 for sample composition) using two distinct methods. First, we estimated the heritability based on the cumulative incidence of MDD as a function of pedigree relatedness (Note S7a). For comparison, the heritability of MDD was subsequently estimated using structural equation modeling in full- and maternal-half sibling pairs.^18^ We fit an ACE model^19^ using maximum likelihood to calculate heritability in OpenMx,^20^ assuming that full-siblings share 50% of their genetic make-up and maternal half-siblings share 25% (A), while both full- and half-siblings fully share their common environment (C) and share no unique environment (E). The models were adjusted for sex, birth year, and birth year squared (Note S7b, Table S6).

### Role of funding source

The authors declare that the funding sources have had no involvement in study design, data collection and analysis, or writing and deciding to submit this manuscript.

## Results

### Global comparisons of socioeconomic indicators and health

**Fig. 2:**
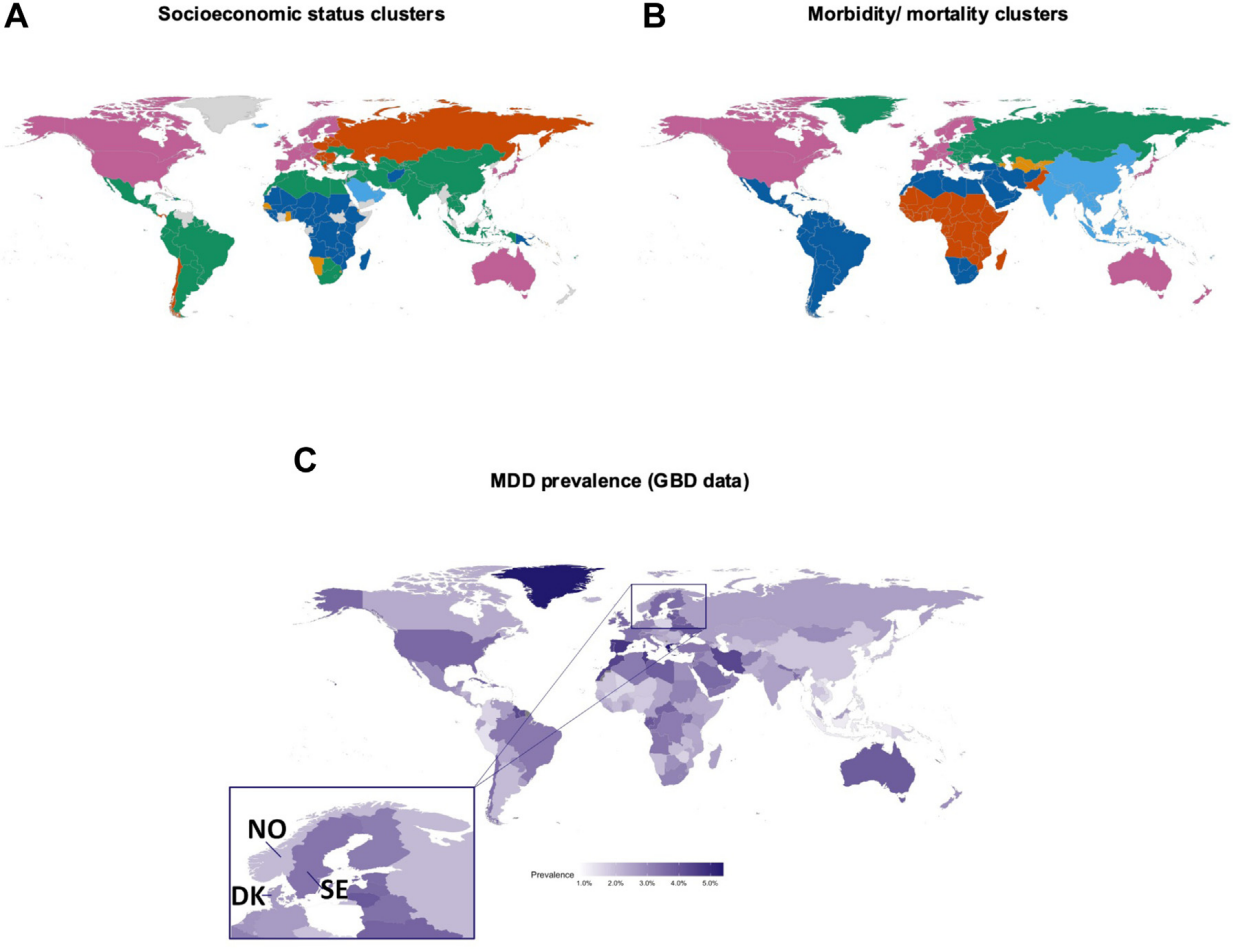
Empirical clustering of global countries. **Panel A)** depicts country clusters with similar key metrics for socioeconomic status. The Scandinavian countries are all in the pink cluster. **Panel B)** shows clustering results on morbidity and mortality, the pink cluster including mostly the same countries as the corresponding socioeconomic cluster from panel A. The bottom **panel C)** shows global prevalence estimates for MDD in 2019, zoomed in on Scandinavia in the inset. NO = Norway; SE = Sweden; DK = Denmark.

**Fig. 3:**
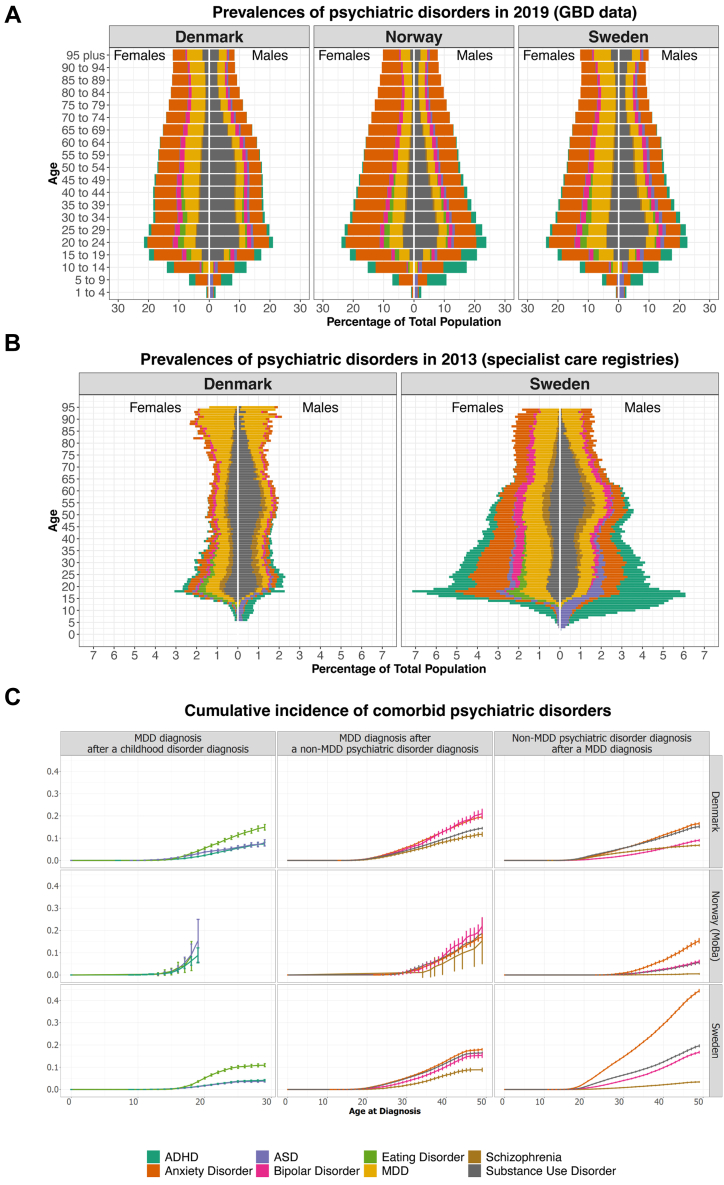
Pyramid plots of the number of diagnoses per age stratum in 2013 and 2019. **Panel A)** represents the number of diagnoses in Denmark, Norway, and Sweden according to the harmonized GBD data. **Panel B)** shows the prevalence in Denmark and Sweden, based on data from specialist care registries. Note that these are counts of diagnoses rather than individuals: people could be counted several times for different diagnoses. **Panel C)** shows the cumulative incidence of receiving an MDD diagnosis after a childhood-onset psychiatric disorder (left column), MDD diagnosis after initial psychiatric disorder diagnosis (middle column) and receiving an additional diagnosis after being diagnosed with MDD (right column). The top row panels show data from the Danish general population, the central row panels represent the MoBa subsample from Norway, and the bottom row panels show results from the Swedish general population. ADHD = attention deficit hyperactivity disorder; ASD = autism spectrum disorder.

The clustering analyses of governmental health care expenditure, GDP, and years of education suggested a high comparability between the Scandinavian countries and other Nordic countries (Estonia, Finland), western Europe (Austria, Belgium, Czechia, France, Germany, Ireland, Italy, Luxembourg, Malta, Netherlands, Portugal, Slovenia, Spain, Switzerland, UK), a few Pacific nations (Australia, Japan, South Korea), North America (Canada, United States), and Israel (Fig. 2A, Table S7, Fig. S1). There were similar clusters for morbidity and mortality rates, with Scandinavia clustering with Nordics (Finland, Iceland), much of Europe (Andorra, Austria, Belgium, Cyprus, France, Germany, Greece, Ireland, Italy, Luxembourg, Malta, Monaco, Netherlands, Portugal, San Marino, Spain, Switzerland, UK), a few Pacific nations (Australia, Japan, New Zealand), and North America (Bermuda, Canada, United States; Table S8, Fig. 2B, Fig. S2). Although highly advantaged according to many metrics, the cluster containing the Scandinavian countries was marked by high morbidity and mortality for psychiatric disorders as well as high mortality for specific other disorders, including neoplasms and neurological disorders (Table S8). Similar 1-year prevalence estimates of MDD were found in this cluster of countries, with North America 3.5%, western Europe 3.9%, and Scandinavia 2.6–3.6% (Fig. 2C). In Scandinavia, prevalence estimates of common psychiatric disorders stratified on age and sex showed similar patterns (Fig. 3A).

### Aim 1. Prevalence of MDD and comorbidity in specialist care

The proportion of the total population diagnosed with MDD in specialist care was 3.1% in Sweden, 3.2% in Denmark, and 4.4% in Norway-MoBa (after exclusions; Table S4). These rates were significantly different, *Χ*^2^ = 1474, *p* < 0.001). Across the countries females were almost twice as likely as males to get a lifetime diagnosis of MDD in specialist care (Relative Risk [RR] = 2.0 in Denmark, RR = 1.3 in Norway, and RR = 1.7 in Sweden). The cumulative incidence by age 49 was 5.4% in Sweden, 6.7% in Denmark, and 9.0% in Norway-MoBa (Fig. S3). In Norway, the cumulative incidence before age 40 was less steep than in Sweden and Denmark, whereas after age 40 it accelerated, which is likely due to the reliance on the MoBa sub sample in Norway (Note S3). Of all ICD-10 MDD diagnoses, a moderate single episode (F32.1) was diagnosed the most frequently in all countries (Table S9). The average age at first specialist diagnosis was slightly higher in Denmark (M = 28.1, SD = 0.04) than in Sweden (M = 26.7, SD = 8.2; *t* = 56.0, *p* < 1E-5, see Fig. S4). Men showed an evenly distributed age of first specialist diagnosis with a median around 27–29 years, and females showed two peaks around 20 and 30–35 years.

To compare the prevalence of MDD to other common disorders, we extracted the most diagnosed psychiatric disorders in the specialist care registers. Overall, MDD, anxiety disorders, substance use disorders and ADHD were the most diagnosed disorders across the different countries (Fig. 3B, Fig. S5–S6). The prevalence of psychiatric disorders was lower in the Danish specialist care register, and the age distribution of MDD appeared different than in Sweden, such that more MDD diagnoses were made in older populations (Fig. 3B).

Psychiatric comorbidity patterns were similar in Denmark, Sweden, and Norway (MoBa), with up to 5–15% developing MDD following a disorder usually developing during childhood (ADHD, ASD, or eating disorders; Fig. 3C). Between 9 and 25% of individuals diagnosed with other (adult-onset) psychiatric disorders were later diagnosed with MDD (before age 50), with anxiety and bipolar disorder being the most prevalent comorbid disorders. In the other direction, the diagnosis of an anxiety disorder after an MDD diagnosis was more than 2.5 times as common in Sweden than in the other two countries (SE: 44.4%, DK: 16.5%, NO: 15.4%). Due to the late start of the patient register in Norway, age at onset of the adult-onset disorders is likely biased upwards in our results (Note S3). We found high rates of several comorbid psychiatric disorders also in a younger cohort of MDD, with consistent patterns in all three countries (Fig. S5, Fig. S6).

### Aim 2. Outcomes and treatment

#### Clinical outcomes

**Fig. 4:**
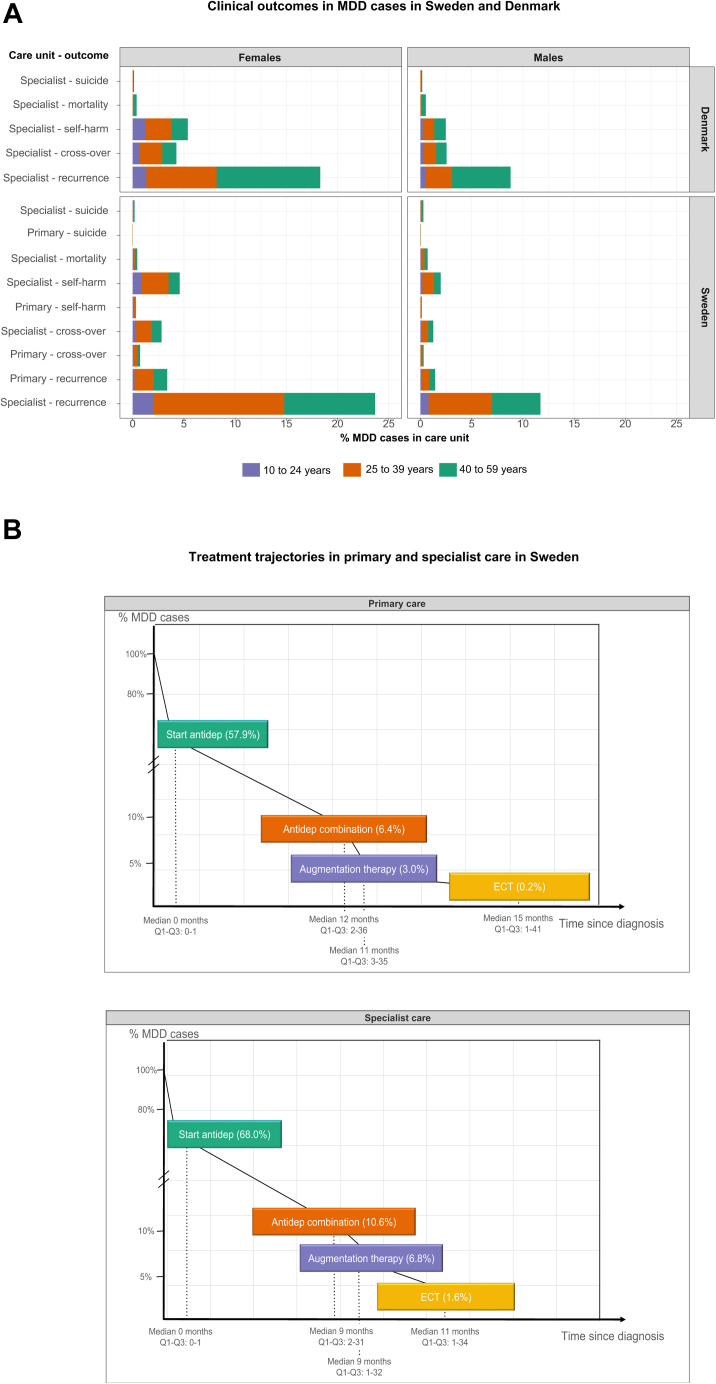
**Panel A)** Prevalence of clinical outcomes as a proportion of all (male and female) MDD cases, split out by specialist and primary care (in Sweden, Stockholm county), cohort of first age at diagnosis, and sex. **Panel B)** Illustration of treatment trajectories in Sweden for primary care (Stockholm County, top panel) and specialist care (bottom panel) with intensification steps as a percentage of the total number of MDD cases. Note that the y-axis excludes the range from 20 to 70% due to the rareness of treatment intensification. Median time to each increasingly intensive treatment step is given with the interquartile range (not scaled). Suicide = death by suicide; mortality = death by non-suicide cause before age 50; recurrence = recurrent MDD as coded in ICD-10; cross-over = development of bipolar disorder, schizophrenia, or schizoaffective disorder after MDD diagnosis; Antidep = antidepressant treatment; ECT = electro-convulsive therapy.

About a third (31%) of MDD cases in specialist care in Sweden and Denmark are diagnosed with recurrent MDD. About 5% ‘cross-over’ to bipolar disorder or schizophrenia/schizoaffective disorder, 7% present with self-harm, 0.4% die of suicide, and 1% die of a different cause (before age 50; see Fig. 4A). Rates for these outcomes were lower for cases in primary care in Sweden (Stockholm county), between 4.8% for recurrence and 0.1% for suicide death. Chi-squared tests for the difference in clinical outcome rates in Sweden and Denmark were not significant (Table S10a). Based on meta-regression results, patterns across age appeared similar between countries (all Cochran's Q *p*-values > 0.05, Table S10b). Most outcomes were more prevalent in females in both countries, except for death by suicide and other-cause mortality, which were more common in men. Self-harm was disproportionately high in young females in both countries. Cross-over was more common in males in Denmark, but more common in females in Sweden. Although for the other outcomes the direction of the sex difference was the same, sex effects were significantly different (all Q *p*-value < 0.005) between the countries for recurrence, mortality, and self-harm, which is probably a result of the large sample size (Table S10b).

#### Socioeconomic outcomes in Sweden

Individuals with a diagnosis of MDD had a significantly higher number of sick leave days in the year after receiving their first diagnosis (M = 53.6 [SD = 109.7]) compared to non-cases in any random year (M = 3.9 [SD = 23.3]; R^2^ = 12.3%; Table S11). MDD cases were more likely to have received government benefit payment for illness or unemployment (3.8%) than non-cases (1.0%; R^2^ = 0.44%). They had a higher yearly income (across 1993–2013, the difference with yearly average in Swedish kronor ×1000: M = 28.4 [SD = 72.7] in cases, and M = −45.8 [SD = 105.0] in non-cases; R^2^ = 0.12%). Cases had a lower educational attainment (M = 370 [SD = 118], on a scale from 100 to 640, falling between upper- and post-secondary education) than non-cases (M = 396 [SD = 114]; R^2^ = 0.30%). For ease of interpretation, we subsequently dichotomized the continuous outcomes and visualized them in Fig. S7 (Note S5). Of note, for dichotomized income the results were flipped, such that MDD cases were more likely to have a low income (>1 SD below average) at least once in the measurement years (25.4% of cases, 17.5% of non-cases; R^2^ = 0.63%). From the figure it appears that the impact of MDD on socioeconomic outcomes was stronger for females; thus, we added interaction terms for MDD and sex. For all outcomes, the interactions were statistically significant, but explained less than 0.1% of the variance. Full regression results can be found in Table S11.

#### Treatment usage: primary and specialist care

In Sweden (Stockholm County), MDD cases had a median of 3 contacts in primary care for MDD (interquartile range [IQR] = 5, N = 39,186). Of those treated in primary care, 33.9% were referred to specialist care. When looking at all MDD cases in specialist care in Sweden, 26.9% had only one outpatient treatment contact, and the median was 3 contacts (IQR = 6). In Demark, specialist treatment use was lower, with median = 1 (IQR = 1, N = 43,699) and 64.5% of patients visiting specialist care only once. In Sweden, females had more treatment contacts than males in primary care (M_difference_ = 1.1 contacts, independent samples t-test *p* = 5.43E-6) while there was no difference in specialist care (M_difference_ = 0.1, *p* = 0.914). In Denmark, females had slightly more treatment contacts than males in specialist care (M_difference_ = 0.2, *p* = 1.1E-28; Fig. S8). Regarding inpatient care in Sweden, 21.0% of cases in specialist care were hospitalized with a main diagnosis of MDD at least once (after the start of the outpatient register in 2001). In Denmark, 31.4% in the specialist care register had one or more inpatient contact (after the start of the outpatient register in 1995). Here we could furthermore derive length of stay, which had a median of 43.4 days (non-consecutive; IQR: 49 days).

#### Pharmacological treatment

In Sweden, 58% of MDD cases used an antidepressant if treated only in primary care, compared to 68% of cases in specialist care (Fig. 4B). Frequently used antidepressants were SSRIs and SNRIs (55% and 22% of all prescriptions, respectively). On average, antidepressants were used for 12.5 months (SD = 9.6, median = 10.4 months, IQR = 9.4 months, N = 14,486), distributed over an average of five treatment episodes (SD = 3.0, median = 4, IQR = 5). They tried on average 2.5 different medications (SD = 1.6, median = 2, IQR = 2; Fig. S9). In specialist care, 11% of cases used ≥2 antidepressants concurrently or received an augmentation from a different medication group. Progression to receiving electro-convulsive therapy (ECT) was rare (<2%).

In Norway, aggregated data for the entire population were available to provide an overview of pharmacotherapy (Note S6). Most antidepressant prescriptions for mood disorders were made in primary care (Fig. S10). On average, 5.4% of females in the general adult population and 3.0% of males were prescribed an antidepressant for mood disturbances in primary care each year. Fewer used antidepressants prescribed in specialist care with 0.54% of males and 0.78% of females in the adult population receiving a prescription for the same indication each year. Overall, there were few fluctuations in use rates, and the sex difference was stable over time.

### Aim 3. Genetic epidemiology

**Fig. 5:**
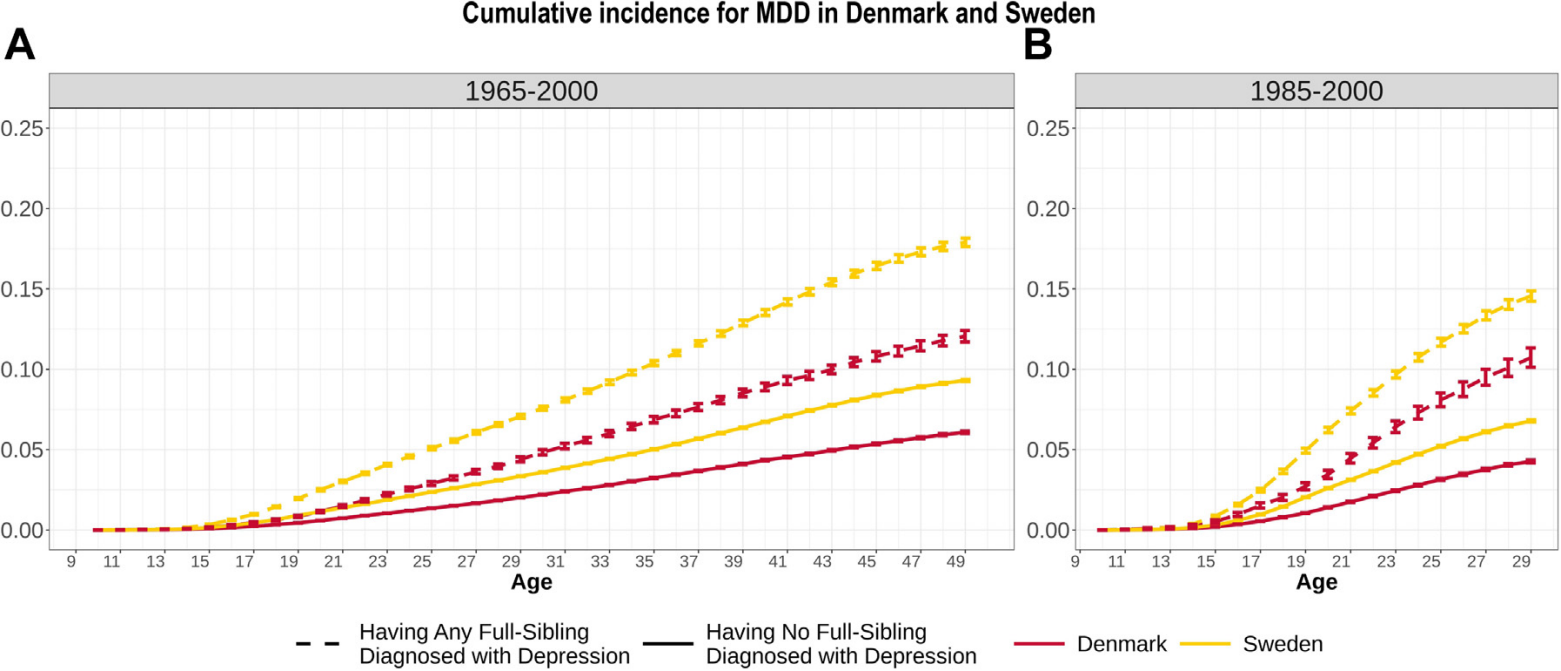
Cumulative incidence for MDD in Denmark and Sweden split out for individuals with and without a full-sibling diagnosed with MDD, separately for the broad (left panel) and narrow birth cohort (right panel).

In order to assess the genetic contribution to MDD we estimated and compared the cumulative incidence for individuals with and without family members with MDD in two different birth cohorts. The cumulative incidence of MDD in the general population by age 49 (for the broad birth cohort 1965–2000) and 29 (for the narrow birth cohort 1985–2000) was estimated at 4.8% (CI = 4.7–4.9%) and 6.8% (CI = 6.7–6.9%) in Denmark, which was significantly lower than the estimates observed in Sweden (7.2%, CI = 7.2–7.3% and 9.8%, CI = 9.7–9.9%; Fig. 5). Having a full-sibling diagnosed with MDD increased the probability of an MDD diagnosis by about 2-fold compared to the risk of MDD in the general population in both countries and birth cohorts (Fig. S11; Table S12A).

We observed in the broad birth cohort that the cumulative incidence of MDD for maternal half-siblings was significantly higher in both countries (*p*_DK_<5.15 × 10^−4^, *p*_SE_<3.81 × 10^−8^) than for full-siblings. This is surprising, given half-siblings are less genetically similar than full-siblings (25% versus 50%) and we assume that they generally share 100% of their family environment (like full-siblings). However, our results showed that in both cohorts, half-siblings had a higher baseline MDD incidence (i.e., without diagnosed siblings) than full-siblings and than the general population (Fig. S12). This is likely due to environmental reasons (e.g., parental divorce or year of birth effects) rather than a biological reason. The cumulative incidence in individuals who have no full-siblings diagnosed with MDD was significantly lower in both countries compared to the risk in the general population. All statistical comparison results within- and between country cumulative incidences are listed in Table S12A–C. In the broad birth cohort, the weighted mean heritability based on cumulative incidences was 40% in Denmark and 47.7% in Sweden. In the narrow birth cohort, the estimates were more similar with 47.2% in Denmark and 48.2% in Sweden.

Heritability estimates based on structural equation modeling, which utilizes exclusively case–control data and no time estimates drawn from a cumulative incidence, were again similar (34.7% and 40.7%, respectively) with lower estimates in the broad cohort than in the narrow cohort (41.9% and 47.7%, Table S13).

## Discussion

Globally, depressive disorders account for the largest proportion of psychiatric disorder-related disability-adjusted life years of all psychiatric disorders.^12^ Broad epidemiological studies are crucial to provide the groundwork for future studies, prevention, and intervention policies. This study is the first effort to present a complete overview of prevalence, outcomes, treatment, and genetic epidemiology across Scandinavia.

The Scandinavian countries cluster together with other western countries and are marked by a high morbidity for psychiatric disorders as well as a high socioeconomic status and healthcare expenditures. This shows that our study results should be broadly generalizable within and outside of Scandinavia. Healthcare is organized in a similar fashion in Sweden, Norway, and Denmark. The prevalence of MDD is accordingly comparable across Scandinavia and to other high-income countries. It is moderately prevalent in Scandinavia, second only to all anxiety disorders combined. Females were affected almost twice as often as males. This sex imbalance is widely known and may be rooted in both biological and sociocultural differences.^21^ We find similar age at first specialist-diagnosis in Denmark and Sweden, with an average of 31 years. The occurrence of comorbid psychiatric disorders before and after first MDD diagnosis was substantial and followed similar patterns across the countries. This aligns with previous findings from the Danish registries, showing enduring, bidirectional comorbidity between mood disorders and other disorders.^22^ Regarding genetic epidemiology, we found a substantial heritability of 35–48%, aligning with previously reported estimates.^23^

Besides these similarities there were also some important differences. Most notably, the prevalence of most psychiatric disorders, comorbidity patterns (with anxiety disorder), occurrence of certain clinical outcomes, and treatment density were lower in the specialist care registry in Denmark than in Sweden. This may reflect differences in diagnostic practice, paths to care and differences in the register infrastructure. In Norway and Denmark healthcare is more centralized, whereas in Sweden counties are largely responsible for providing health care. In Denmark, an individual typically visits their general practitioner (GP) 8 times per year, compared to 3 in Norway and only 1.3 in Sweden. Likewise, more people are treated for a psychiatric disorder in specialist care in Sweden (4.3% of the population in 2013) and Norway (5%^24^) than in Denmark (3.1% in 2013). Furthermore, while a referral from the GP to specialist health care is the most common path in Denmark and Norway, patients often contact specialist care directly in Sweden.^10^ Finally, data from private health care facilities that treat individuals referred by the national systems, have become available at different dates in the countries, which might have contributed to selective underreporting.^25^^,^^26^ In particular, private practitioners in Norway receiving tax funds and all private psychiatrists in Sweden report to the registries, while no private practitioners in Denmark do so. Such discrepancies are reflected in the observed lower rates of specialist care contacts for MDD and other psychiatric disorders in Denmark. Additionally, the lifetime risk of MDD differed in the overall population and by sibling type between Denmark and Sweden. This suggests that besides genetic and common environmental differences, other factors may contribute to the observed difference between sibling types and between countries. Future research should take these differences into consideration when comparing results across Scandinavian countries and should ideally leverage data from both primary and specialist care.

Overall, our results show that MDD is associated with a substantial disease burden. For instance, we observed increased chances of suicide, self-harm and early mortality in both Sweden and Denmark. In Sweden, we showed that MDD was associated with less favorable socioeconomic outcomes, and this was especially the case for females. Such inequalities might work to exacerbate MDD disease burden and might be even more pronounced in other countries.^27^ It needs to be pointed out that the majority of MDD cases live in countries that are not comparable to Scandinavia in terms of socioeconomics and morbidity/mortality according to our clustering analysis. Future research should expand to low/middle income countries to identify targets for decreasing health inequities in MDD.^13^ The occurrence of treatment combinations and augmentation trajectories suggest that, for many patients, initial treatment fails to resolve MDD symptoms.^28^ For instance, 1.6% of cases in specialist care in Sweden go on to receive ECT-treatment. These findings show that MDD can develop into a severe or treatment-resistant condition, and confirm that the disease burden of MDD is incessantly high.^13^

It is important to note the strengths and limitations of our analyses. This is the first study to provide a comprehensive, in-depth overview and country comparison of the epidemiological trajectories of MDD. We relied on high quality data from two broad sources: public repositories and national registers capturing real-world, clinically recorded MDD. The national registers are goldmines for population studies, given their nationwide coverage of multiple domains across several decades. The timelines of the registers differ but have been available for the greater part of the last five decades. We showcased how to mine and compare these registers across countries. The drawback of this comprehensive approach was that we could not go into detail on each of the different aspects, so that our results do not encompass topics such as symptom severity, pharmacological treatment adherence or psychological treatment procedures. As a more specific limitation, the Norwegian register data were available only for the MoBa cohort, which is consent-based and thus has some selection bias.^16^ In addition, the Norwegian patient register only covers the period from 2008 onwards (for this study 2008–2018), thereby censoring the young adulthood for the majority of MoBa participants. Still, we found the prevalence of MDD to be comparable to the nationwide Swedish and Danish register data. Another limitation is that data from before the 2000s derives mostly from more severe inpatient cases. In Sweden, ICD-codes of psychiatric diagnoses in outpatient clinics were underreported until around 2005,^29^ which could explain the difference seen in prevalence of MDD in Sweden before and after excluding individuals who received their diagnosis less than 5 years before 2013. Also, in our comparisons we did not address the potential within-country differences. This limits generalizability; geographical differences in the use of health care services are well known.^30^^,^^31^ We observed high rates of psychiatric comorbidity. Due to the heterogeneity in MDD, this could reflect diagnostic uncertainty rather than actual comorbidity. We aimed to account for this possibility by looking at comorbidity patterns over time, and when looking at diagnostic cross-over as a clinical outcome we applied a wash-out period (Note S4g and S5a). A final limitation was that our study population was limited in diversity due to our exclusion of individuals born outside of the country (in Sweden and Denmark). Future research should expand to include such groups and identify potentially group-specific targets for prevention and intervention.

The current findings of key epidemiological characteristics of MDD across Scandinavian countries provide clues for venues to explore in future research. This study maps the moderate heritability, high prevalence, debilitating clinical features, high comorbidity, poor outcomes, and high treatment use associated with MDD in the healthcare systems of Scandinavia. It provides key information for researchers seeking to use these registers. Our findings emphasize the need for prevention and intervention and can act as a starting point for identifying targets for policy making regarding clinical strategies.

## Contributors

JAP, JJM, and MH were the main analysts in Sweden, Denmark, and Norway, respectively. They led the drafting of the manuscript and created the figures and had equal contributions. AH, YX, TDN, AJ, PFS, and MT performed additional statistical analyses. PL, HL, TW, TRK, and OA were crucial in acquiring funding and data access. IO and RZ assisted with data curation. KK, JRS, JB, UD, and AT acted as consultants in the writing and analysis process. PFS, KK, AB, MT, and YL were responsible for the conceptualization of the study and supervised the analysis and writing process. AB, MT, and YL had equal contributions. All authors have contributed in reviewing and editing the manuscript.

## Data sharing statement

This work is based on Scandinavian register data that cannot be shared in any form, to comply with privacy guidelines as stated in the General Data Protection Regulation (GDPR). Information on acquiring data access can be acquired at https://www.dst.dk/en/TilSalg/Forskningsservice (DK), https://www.socialstyrelsen.se/en/statistics-and-data/statistics/ (SE), and https://www.ssb.no/en/data-til-forskning/utlan-av-data-til-forskere (NO), or by contacting the senior corresponding authors.

## Ethical approval

The use of Danish data was approved by the Danish Health Data Authority (project no. FSEID-00003339) and the Danish Data Protection Agency. By Danish law, informed consent is not required for register-based studies. The study was approved by the regional ethics review board in Stockholm, Sweden. The establishment of the MoBa cohort and initial data collection was based on a license from the Norwegian Data Protection Agency and approval from The Regional Committees for Medical and Health Research Ethics, and the MoBa cohort is now following the regulations of the Norwegian Health Registry Act. The current study was approved by the administrative board of the Norwegian Mother, Father and Child Cohort Study led by the Norwegian Institute of Public Health and The Regional Committees for Medical and Health Research Ethics (2016/1226/REK sør-øst C) in Norway.

## Editor note

The Lancet Group takes a neutral position with respect to territorial claims in published maps and institutional affiliations.

## Declaration of interests

PFS is a scientific advisor and shareholder for Neumora Therapeutics. AJ has served as a speaker for Takeda. HL reports receiving grants from Shire Pharmaceuticals; personal fees from and serving as a speaker for Medice, Shire/Takeda Pharmaceuticals and Evolan Pharma AB; and sponsorship for a conference on attention-deficit/hyperactivity disorder from Shire/Takeda Pharmaceuticals and Evolan Pharma AB, all outside the submitted work. HL is editor-in-chief of JCPP Advances. OAA is a consultant to HealthLytix and received speaker's honorarium from Sunovion and Lundbeck.

## Appendix A Supplementary data

Supplementary Notes S1–S7

## References

[1] Günther T., Malmström H., Svensson E.M. (2018). Population genomics of Mesolithic Scandinavia: investigating early postglacial migration routes and high-latitude adaptation. PLoS Biol.

[2] Lao O., Lu T.T., Nothnagel M. (2008). Correlation between genetic and geographic structure in Europe. Curr Biol.

[3] Margaryan A., Lawson D.J., Sikora M. (2020). Population genomics of the Viking world. Nature.

[4] Warner-Søderholm G. (2012). But we’re not all Vikings! Intercultural identity within a nordic context. Intercult Commun.

[5] Beugelsdijk S., Welzel C. (2018). Dimensions and dynamics of national culture: synthesizing hofstede with inglehart. J Cross Cult Psychol.

[6] Helliwell J.F., Layard R., Sachs J.D., de Neve J.-E., Aknin L.B., Wang S. (2021). World Happiness Report 2021. https://happiness-report.s3.amazonaws.com/2021/WHR+21.pdf.

[7] World Bank (2020). https://data.worldbank.org/indicator/NY.GDP.PCAP.CD?contextual=max&end=2020&locations=SE-NO-DK&name_desc=true&start=1960&type=shaded&view=chart&year=2020.

[8] Organisation for Economic Co-operation and Development (2021). https://data.oecd.org/healthres/health-spending.htm.

[9] OECD (2021).

[10] Laugesen K., Ludvigsson J.F., Schmidt M. (2021). Nordic health registry-based research: a review of health care systems and key registries. Clin Epidemiol.

[11] World Health Organization (1992).

[12] GBD 2019 Mental Disorders Collaborators (2022). Global, regional, and national burden of 12 mental disorders in 204 countries and territories, 1990–2019: a systematic analysis for the Global Burden of Disease Study 2019. Lancet Psychiatry.

[13] Herrman H., Patel V., Kieling C. (2022). Time for united action on depression: a lancet–world psychiatric association commission. Lancet.

[14] Ritchie H. (2019). https://ourworldindata.org/12-key-metrics.

[15] McInnes L., Healy J., Melville J. (2018).

[16] Magnus P., Birke C., Vejrup K. (2016). Cohort profile update: the Norwegian mother and child cohort study (MoBa). Int J Epidemiol.

[17] Athanasiadis G., Meijsen J.J., Helenius D. (2022). A comprehensive map of genetic relationships among diagnostic categories based on 48.6 million relative pairs from the Danish genealogy. Proc Natl Acad Sci U S A.

[18] Rijsdijk F.v., Sham P.C. (2002). Analytic approaches to twin data using structural equation models. Brief Bioinformatics.

[19] Maes H.H. (2005). Encyclopedia of statistics in behavioral science.

[20] Boker S., Neale M., Maes H. (2011). OpenMx: an open source extended structural equation modeling framework. Psychometrika.

[21] Altemus M., Sarvaiya N., Neill Epperson C. (2014). Sex differences in anxiety and depression clinical perspectives. Front Neuroendocrinol.

[22] Plana-Ripoll O., Pedersen C.B., Holtz Y. (2019). Exploring comorbidity within mental disorders among a Danish national population. JAMA Psychiatry.

[23] Kendall K.M., van Assche E., Andlauer T.F.M. (2021). The genetic basis of major depression. Psychol Med.

[24] Tesli M.S. (2021). https://www.fhi.no/en/op/hin/mental-health/psykisk-helse-hos-voksne/.

[25] Schmidt M., Schmidt S.A.J., Sandegaard J.L., Ehrenstein V., Pedersen L., Sørensen H.T. (2015). The Danish National Patient Registry: a review of content, data quality, and research potential. Clin Epidemiol.

[26] Socialstyrelsen (2022).

[27] Assari S. (2017). Social determinants of depression: the intersections of race, gender, and socioeconomic status. Brain Sci.

[28] Fabbri C., Corponi F., Souery D. (2019). The genetics of treatment-resistant depression: a critical review and future perspectives. Int J Neuropsychopharmacol.

[29] Socialstyrelsen (2014).

[30] Surén P., Thorstensen A.G., Tørstad M. (2018). Diagnostikk av hyperkinetisk forstyrrelse hos barn i Norge. Tidsskr Nor Legeforen.

[31] Johansson N., Jakobsson N., Svensson M. (2018). Regional variation in health care utilization in Sweden – the importance of demand-side factors. BMC Health Serv Res.

