# Supplementary material for: Epidemiological overview of major depressive disorder in Scandinavia using nationwide registers
Source: Lancet Reg Health Eur. 2023 Mar 28;29:100621. doi: 10.1016/j.lanepe.2023.100621 (PMC10230616; doi:10.1016/j.lanepe.2023.100621)
Supplement: Supplementary Notes S1–S7 [file mmc1.docx]

**Supplementary notes**

[1. Global comparisons of socioeconomic indicators and health 2](#_Toc127024851)

[2. Study population: data from the national registers 3](#_Toc127024852)

[3. Data from the Norwegian Mother, Father and Child Cohort Study (MoBa) 4](#_Toc127024853)

[4. Aim 1. Prevalence of MDD and comorbidity in specialist care (Norway (MoBa), Denmark & Sweden) 4](#_Toc127024854)

[5. Aim 2a. Clinical (Sweden & Denmark) and socioeconomic outcomes (Sweden) 5](#_Toc127024855)

[6. Aim 2b. Treatment usage in primary and specialist care 7](#_Toc127024856)

[7. Aim 3. Genetic epidemiology (Sweden & Denmark) 9](#_Toc127024857)

[References 11](#_Toc127024858)

### Global comparisons of socioeconomic indicators and health

We wished to understand the relative places of DNK, NOR, and SWE in comparison to other countries in the world. The reason is because the generalizability of findings from our project to other countries depends strongly on the results – do the Nordic countries stand apart even with respect to other countries in northwest Europe? Or are they comparable to countries with similar economies and health metrics? We evaluated these questions using two broad sets of data for the countries of the world: ‘key metrics’ for demography, gross domestic product, fertility, life expectancy, education, and infrastructural features (access to sanitation, clean water, and electricity); and morbidity and mortality across many types of diseases (including mental disorders).

Key metrics. We used the ‘12 key metrics’ from Our World in Data (<https://ourworldindata.org/12-key-metrics>^1^, see Table S2; please see the web page for full details). We were able to compile complete data for 168 countries for 11 metrics: population, life expectancy at birth, gross domestic product per capita, mean years of schooling, childhood mortality <5, fertility per woman, government spending on health care (fraction of total spending), energy use per capita, and access to sanitation, clean water, and electricity (fraction of population). Almost all data were from 2019. For a few measures where 2019 data were missing, we brought forward data from the nearest non-missing year for that country. All data are continuous.

Burden of disease. We obtained morbidity and mortality data for 204 countries from the 2019 Global Burden of Disease Study (<https://ghdx.healthdata.org/gbd-results-tool>^2^, see Table S3). Mortality rates per country were for the GBD Level 2 categories: tropical diseases and malaria, nutritional deficiencies, neoplasms, cardiovascular disease, chronic respiratory disease, digestive disease, neurological disease, mental disorders, alcohol use disorders, drug use disorders, musculoskeletal disease, other non-communicable disease, skin disease, transportation injury, unintentional injury, self-harm and interpersonal violence, HIV/AIDS and sexually transmitted infection, respiratory infections and tuberculosis, enteric infection, maternal and neonatal disease, and diabetes and renal disease. Morbidity rates per country were for Level 1 categories for most types of disease and detailed categories for psychiatry disorders (for most of these, only morbidity data are available): communicable disease, maternal and neonatal disease, and nutritional deficiency; non-communicable disease; unintended injury; self-harm and interpersonal violence; and MDD, bipolar disorder, anxiety disorders, schizophrenia, eating disorders, alcohol use disorders, and drug use disorders.

Data visualization. We applied the following steps separately to the key metric and disease burden data: (a) all of these continuous variables were standardized (i.e., mean of 0 and standard deviation of 1), and no variables were highly correlated within each data frame; (b) we applied UMAP to reduce data complexity to two dimensions (this choice is standard); (c) applied HDBSCAN to detect clusters; and then (d) plotted and analyzed the results. Default parameters were used.

Prevalence of MDD and other psychiatric disorders: Prevalence estimates from GBD were retrieved from <https://vizhub.healthdata.org/gbd-results/>. First, we included data for 204 countries and presented the estimates in a map for 2019, using R package mapproj. Data was retrieved with the search term “Major depressive disorder”, in both sexes and all ages. Second, we obtained prevalence estimates for additional common mental disorders, using the search terms “Autism spectrum disorders” (ASD), “Attention-deficit/hyperactivity disorder” (ADHD), “Anxiety disorders”, “Bipolar disorder”, “Eating disorders”, “Schizophrenia” and “Substance use disorders” in Denmark, Norway and Sweden for 2019, split by sex and age clusters of five years. The diagnoses were renamed to better fit the figure legend. Results were presented in a pyramid plot stratified by age and sex using R-package ggplot2 (see Table S3 for data sources GBD used to derive estimates).

### Study population: data from the national registers

For most analyses, our study relied on data collected in the national registers (see Fig. 1 in main manuscript). We used data from the following registers:

Patient registers: Inpatient data registration started in 1973 in Sweden and 1977 in Denmark, whereas outpatient registration started in 2001 and 1995, respectively. In Norway, in- and outpatient registration both started in 2008. The countries use the ICD classification system from the WHO. For the most recent registry entries, the 10^th^ ICD revision is used, which was introduced in the Scandinavian countries between 1994 and 1999. For older entries, we used the ICD-9 and ICD-8 equivalents (see Table S5 for ICD codes).

Primary care register: primary care register data were available for Stockholm County (capital region of Sweden). This register was started in 2003 and uses ICD-10 diagnostic codes. It includes data on ~2.5 million individuals, which is most of the population living in Stockholm County between 2003-2013.

Cause of death: in order to assess all-cause mortality and suicide in MDD cases we made use of the cause of death registers in Denmark and Sweden. Registrations of suicide are often confirmed by forensic autopsy. Death certification in Scandinavia ranks among the world’s best^3^.

Prescription register: Individual-level registry data on dispensed medications were available in Sweden, with data from 2005-2015, and aggregated country statistics were available in Norway.

Multigeneration register: Pedigree information (used to estimate MDD heritability) was available in Sweden and Denmark and was retrieved from the respective multigeneration registries.

To provide insight on what original and wrangled data looked like, we provide exemplary (bogus) data in Table S14. These data are based on the structure of the Swedish patient register, but other registers from Sweden, Norway, and Denmark are constructed in a similar manner.

### Data from the Norwegian Mother, Father and Child Cohort Study (MoBa)

MoBa is a Norwegian prospective pregnancy cohort, initiated by epidemiologists primarily to investigate pregnancy outcomes. Pregnant women in Norway were invited to participate between 1999-2008. All pregnant women were considered eligible, only restricted to an ability to read Norwegian. By the end of inclusion, 50 of 52 hospitals with maternity units were involved in the study, and the participation rate was 41%. Blood samples were obtained from mother, father and child, and other measurements were based on questionnaires. Included families are still continually asked to answer questions about illnesses and environmental factors related to health. The oldest children have now reached 23 years of age. Over time, multiple sub-studies have evolved. The main aim has been to understand pathogenesis of complex disorders, and the availability of data regarding mental health is vast. For the present study, linked data from the Medical Birth Registry and the National Patient Registry for the included participants were used. For detailed information about MoBa, please see ^4^.

### Aim 1. Prevalence of MDD and comorbidity in specialist care (Norway (MoBa), Denmark & Sweden)

Prevalence:

1. We identified MDD cases by identifying individuals who received at least one ICD8, 9 or 10 diagnosis of MDD (Table S5) in the covered specialist care register period (varying between 1973-2018, see Table S1). The prevalence of MDD was derived as a percentage of the defined background population (Table S1/S4). Specifically, we divided the number of cases in specialist care by the number of individuals in the general population register that were born in the same window as the MDD cases (1944-2009/ 1965-2000). We did this both for the MDD case cohort before and after the analytic exclusions. Multiple exclusions were made to ensure there were follow-up data of at least 5 years for all cases and aimed to provide sufficient data coverage. In the Norwegian MoBa sample, this was not applied, due to a smaller sample size and shorter coverage period available (2008-2018). Due to different register periods the number of excluded individuals differed substantially in the different countries.
2. Because of GDPR regulations, individual-level data were not shared between the countries. Instead, we used aggregated, summary-level data to compare patterns between countries. We created contingency tables counting the number of cases and non-cases per country, stratified by sex. We performed chi-squared tests on these tables to compare the rates between the countries. We did not perform a Yates correction because of the large sample size. We computed the average relative risk of MDD by sex across the countries.
3. The cumulative incidence of MDD was derived separately in each country using sub distribution analysis of competing risk as implemented in the R-package cmprsk, resulting in group level aggregated results. Using this method, we derive the prevalence of MDD at each 1-year age stratum, taking into account censoring and competing risk. We plotted the cumulative incidence as a function of country and age.
4. We derived age at first diagnosis by computing the difference in years between birth year and the year of the first record of MDD in the register. Subsequently, we created a frequency table for Denmark and Sweden and plotted the density of age at first diagnosis and derived medians. We did not include Norway in these descriptions, because of the limited (age) coverage of the MoBa cohort. We computed the mean difference, pooled standard deviation, and an independent samples *t* (assuming equal variances) to test if the mean age was significantly different in Denmark and Sweden.

Comorbidity:

1. To put the prevalence of MDD in specialist care into context, we extracted the prevalence of all mental disorders (ICD-10) in the total specialist care register and compared it to that in people who had at least one registered diagnosis of MDD. We visualized the results in bar graphs S5-S6. In Norway (MoBa), codes that were not available for analyses as well as ICD 10 codes with a count of less than 6 individuals in any of the bars (following GDPR regulations), were shown in strikethrough text.
2. We then selected mental disorders common in MDD cases for follow-up analysis in Sweden and Denmark. Those were autism spectrum disorder (ASD), attention deficit hyperactivity disorder (ADHD), anxiety disorder, bipolar disorder, eating disorder, schizophrenia/ schizoaffective disorder, and substance use disorder. We derived how often these disorders were diagnosed, stratified by sex and age in the respective secondary care registers. As we were interested in age-stratified rates we selected 2013 as a single measurement year (the most recent year for which we had data in all three countries). Using rates of MDD and other disorders we visualized how MDD coincides with and compares to different disorders in a pyramid plot.
3. Next, we assessed common co-morbidity patterns over time for MDD focusing on the cumulative incidence of the selected disorders. We looked at how many individuals with a childhood-onset disorder (ASD, ADHD, or eating disorder) developed MDD before age 30, and how many individuals with a disorder usually occurring in adulthood (anxiety disorder, bipolar disorder, schizophrenia, or substance use disorder) received an MDD before age 50. Reversely, we looked at how many people with MDD developed one of the adult-onset disorders by that age.

### Aim 2a. Clinical (Sweden & Denmark) and socioeconomic outcomes (Sweden)

Clinical outcomes:

1. In Denmark and Sweden, we extracted information on clinical outcomes in the specialist care register. In Sweden, information from primary care in Stockholm was available (N_MDD cases_=48,915). We extracted the first instances of the following binary outcomes in all MDD cases:
   - Recurrence of MDD: having any F33 code (only available in ICD-10)
   - Diagnostic cross-over: having a code for schizophrenia, schizoaffective disorder, or bipolar disorder (see ICD codes in Table S5) after the first recording of MDD. We excluded individuals that crossed over within 6 months after their first MDD diagnosis, as this may represent diagnostic refinement rather than cross-over.
   - Self-harm: any recording of self-harm (see ICD codes in Table S5), excluding those for individuals who had died of suicide to prevent double counting.
   - Suicide: any recording of self-harm as a cause of death in the cause of death register (see ICD codes in Table S5). Note that for suicide we do not use the 5-year follow-up window that we applied for the other outcomes, so that individuals who die by suicide within 5 years after their diagnosis are not excluded.
   - Mortality: all-cause mortality from the cause of death register. Due to the coverage of the register and the selection of the birth cohorts, the maximum age of patients in our cohort was 49. Thus, this variable represents early death.

We aggregated the data and calculated the prevalence of each outcome within the group of cases, stratified according to care unit (specialist versus primary), country (Denmark versus Sweden), sex, and age stratum (10-24, 25-39, and 40-59). We plotted the percentage of MDD cases for whom each of these outcomes was recorded, as a function of these different factors.

1. Next, we compared the rates per country using a chi-squared test for each outcome, without Yate’s correction (which is appropriate in smaller samples). Input for this test was a contingency table for each outcome (present/ absent by Denmark/ Sweden). Using the same input, phi was estimated as a measure of effect size.

To test for sex differences and age effects, we performed logistic regression analysis within both countries. We used age at the end of the register period (2013) to capture age effects. Because this was almost perfectly collinear with age in 2013, we did not include birthyear as covariate. We performed logistic regression separately for each clinical outcome as a dependent variable, and age and sex as independent variables. Subsequently, we performed meta-analysis on the betas and confidence intervals from both countries. We report pooled beta’s and standard errors, and extracted Cochrane’s Q to assess differences in sex and age effects in Sweden and Denmark.

Socioeconomic register outcomes:

1. For Sweden, we were able to furthermore investigate socioeconomic outcomes. We focused on the prevalence of receiving benefit payments, sickness leave, income, and educational attainment. We use data from the longitudinal integrated database for health insurance and labour market studies (LISA) registry from 1993-2013. We excluded measurements when individuals were below age 15. Outcomes were defined as follows:
   - Benefits (binary): we assessed if individuals had received compensation for sickness or unemployment in the 5-year period after their first diagnosis, or within a random 5-year window in non-cases. Individuals were coded with 1 if they received compensation for a period of 14 or more consecutive leave days for sickness or employment injury, or had received any compensation for unemployment.
   - Sickness leave (continuous): we assessed the number of sickness days in the year after the first MDD diagnosis in cases and compared it to the number in a random year in non-cases.
   - Income (continuous): we computed the difference between the yearly disposable income (after taxes, in 1000s of Swedish kronor) and the yearly average, and took the average difference score across the measurement years. Thus, we capture the deviation from the country average over time. Because we were mainly interested in income from other sources than benefit payments (which was covered in another variable) we removed the measurement instance if the person had received benefit payments in that year.
   - Educational attainment (continuous): we treated the six standardized levels with sub levels (100=primary/ lower secondary school to 640=postgraduate, doctoral) as a continuous variable.

For visualization purposes, we dichotomized the continuous measures so that we could plot the proportion of each outcome in MDD-cases versus non-cases (Figure S7). We used the following binary definitions:

- Low income: having an income less than 1 SD below the yearly average for at least one measurement year.
- Low educational attainment: the maximum level of education attained between 1993-2013 fell more than 1 SD below the yearly average for at least one year.
- Many sick days: having more sick days than 1 SD above the yearly average in the year after MDD diagnosis for cases, or more than 1 SD above the yearly average for a randomly selected measurement year for non-cases.

1. We formally tested the effect of MDD status on socioeconomic outcomes while controlling for sex and birth year using regression. We used centered and standardized continuous outcomes with linear regression where available (sickness leave, income, and educational attainment). In a separate step, we added multiplicative interaction terms between MDD status and sex. With adjusted or Nagelkerke’s R^2^ (for continuous or binary outcomes, respectively) we derived the variance explained in the socioeconomic outcomes by MDD and by the interaction between MDD and sex.

### Aim 2b. Treatment usage in primary and specialist care

Treatment use:

1. To assess treatment use in Sweden and Denmark, we extracted the number of treatment contacts in primary and specialist care with MDD as main diagnosis. These contacts will encompass both psychological and pharmacological treatment. No information was available to provide insight into the used treatment methods within the patient registers. Instead, we describe how many patients had only one contact in specialist care, extract the median, and the interquartile range (IQR). The distribution of the number of treatment contacts was plotted in violin plots stratified by sex and country. We removed outliers before plotting the results (>250 visits, N=33) from the Swedish data to ensure readability (Figure S8). Subsequently, we tested sex differences in treatment use, applying a two-sided independent sample t-test within both countries. In Sweden, for each treatment contact it was recorded whether it was an outpatient or inpatient contact. We derived the number of patients that had at least one inpatient contact with a main diagnosis of MDD to capture hospitalization. We considered the period after the start of the outpatient register (2001) because before that, only inpatient records were kept. In Denmark, we followed identical procedures and extracted the number of inpatient contacts after the start of the outpatient register in 1995. We could furthermore extract information on the length of inpatient stay. We counted the total number of days that cases had stayed, without considering whether these were consecutive days, and derived the median and IQR.

Antidepressant use:

Using the Swedish and Norwegian prescription drug registers, we investigated the prevalence of antidepressants use.

1. In Sweden, we extracted rates of use and use patterns for antidepressant medications (coded N06A according to the Anatomical Therapeutic Chemical classification system [ATC]^5^) in MDD cases. We restricted our population to individuals diagnosed with ICD-10 in or after 2006 (at least 5 years after the start of the outpatient register). We applied a washout period of one year, removing individuals that had dispensed an antidepressant in the year before their first MDD diagnosis. We excluded individuals with a diagnosis of schizophrenia or bipolar disorder before the first MDD diagnosis. We derived medication treatment episodes, defined as periods where prescription dates are maximally 4 months apart. If there was a gap of more than 4 months until a new prescription, this was considered a new episode ^6,7^. We report the average duration and number of medication episodes, as well as the number of different antidepressants used by each individual. Furthermore, we explore the occurrence of antidepressant combination (concurrent use of >1 antidepressant medication) and augmentation (concurrent use of antidepressant medication and any of lithium, risperidone, olanzapine, aripiprazole or quetiapine^8^). Concurrent use was defined as redeeming a prescription for different medications within 14 days. We derived the duration of combination and augmentation treatment by extracting the overlapping time between initiated antidepressant use and the use of antidepressant combination or augmentation.

To provide insight in which antidepressants and which combinations were the most commonly prescribed for MDD, we created an upset (‘piano’) plot (Fig. S9). We extracted the number of cases that had used one or any combination of compounds from four different categories over the course of the covered register period. The upset plot provides counts the occurrence of different combinations of mood stabilizer, antipsychotics, SSRI, and other antidepressant usage.

1. In Norway, we used aggregated country level data and extracted rates of antidepressant use in both primary and secondary care for the population aged 18-64 years, not restricted to MDD cases. Due to the aggregated nature of the data, one individual could be counted in both primary and secondary care the same measurement year. The background population was calculated as the mean population size in each measurement year (i.e. the average of the population January 1^st^ and December 31^st^). We report rates where the clinical indication for the prescribed antidepressant (ATC group N06A) was collected from the prescription, and mainly based on reimbursement codes -73 (ICPC) or -F3 (ICD) with the designation ‘Mood disturbances in need of treatment’. In some cases, the clinical indication for prescription was F32/F33 (ICD-10) in specialist health care or P76 (ICPC) in primary care. Antidepressant use was presented stratified for age and sex in Fig. S10.
2. In Sweden, we could provide an overview of common treatment trajectories. Within both care units, we could derive how many cases start with an antidepressant. Of the people who had started, we derived how many progressed to receiving treatment combination and augmentation. Also, we extracted the median and IQR for the time between the initiation of antidepressant treatment to this treatment intensification step. Next, we derived how many of the cases who received augmentation therapy would subsequently receive ECT treatment. In Sankey plots (Fig 4) we present how many cases go through these treatment intensification steps, and we give the median time (with IQR) until a next step is taken.

### Aim 3. Genetic epidemiology (Sweden & Denmark)

For estimating the heritability of MDD we used diagnostic status in the specialist care register in sibling pairs. Each ascertained individual was assigned as either 0 = a censored case: individuals never diagnosed with MDD and lived past the end of the follow up period or emigrated before said period; 1 = an MDD case: individuals with an MDD diagnosis; or 2 = a competing risk case: individuals never diagnosed with MDD and who had died before the end of follow up. All analyses were performed using full-siblings (50% genetically similar and mostly sharing an environment) and maternal half-siblings (25% genetically similar and generally sharing an environment). An overview of the number of samples used for the genetic epidemiology analyses is given in Table S6.

1. Cumulative incidence

First, we derived the heritability based on the cumulative incidence of MDD as a function of pedigree relatedness following procedures described by Wray and Gottesman^9^. For each individual, the time to event in years, given their assigned status, was calculated as: (0) age at end of follow-up or emigration, (1) age at first MDD diagnosis, and (2) age at death. The incidence of MDD in both countries was calculated based on the number of new individuals diagnosed with MDD for each one-year increment in age (represented by the time to event). Cumulative incidences were estimated using the Nelson-Aalen estimator, which can incorporate censored and incomplete data. The cumulative incidence represents the proportion of individuals in the population that have or will be diagnosed with MDD up to a given age. Here we calculated the cumulative incidences of MDD using the Danish and Swedish registers for: a) the general population (all individuals without restrictions on competing diagnoses and relatedness), b) individuals with one or more full-siblings/maternal half-siblings diagnosed with MDD, and c) individuals with no full-siblings/maternal half-siblings diagnosed with MDD. Based on these cumulative incidences the additive heritability of MDD under the liability threshold model can be estimated. We obtain overall estimates per country by weighting the full-sibling and maternal half-sibling estimates by the inverse of their sampling variances. Note that due to the observed inflation in maternal half-siblings cumulative incidences (Table S12) we opted for P(MDD given no affected maternal half-siblings) as baseline depression risk for all estimates (instead of general population estimates). Differences between cumulative incidences, between and within countries, were assessed by calculating the *z*-score (see formula below) and corresponding *p*-value using estimates by age 49 (for the broad birth cohort 1965-2000) and 29 (for the narrow birth cohort 1985-2000). Statistical significance was determined via a conservative Bonferroni correction (0.05/number of comparisons).

$$z= \frac{{Estimate CIF}_{1}-{Estimate CIF}_{2}}{\sqrt{{SE}_{Estimate CIF1}^{2}+{SE}_{Estimate CIF2}^{2}}}$$

Estimate CIF = Cumulative incidence of MDD by age 49 or 29, SE = Standard error

1. Structural equation modeling

Next, the heritability of MDD on the liability scale was estimated from full-sibling and maternal half-sibling pairs using structural equation modeling in OpenMx^10^. We selected sibling pairs born within the selected cohort and within 10 years of each other. To avoid underestimating the standard error by including pairs from the same family, we randomly selected one pair per family. As the outcome was a binary variable, we incorporated the liability threshold model to estimate the heritability on liability scale ^11^. We fit an ACE model to calculate heritability, assuming that full-siblings share 50% of their genetic make-up while maternal half-siblings share 25% (A), while both full- and half-siblings fully share their common environment (C) and share no unique environment (E). The total liability of MDD comes from A, C and E, the variance of the phenotype and covariance of the phenotype between siblings could be decomposed into 3 equivalent components as:

For full siblings:

$\left| \begin{matrix} var & covar \\ covar & var \end{matrix} \right|$=$\left| \begin{matrix} a^{2}+c^{2}+e^{2} & {0.5a}^{2}+c^{2} \\ {0.5a}^{2}+c^{2} & a^{2}+c^{2}+e^{2} \end{matrix} \right|$

For maternal half-siblings:

$\left| \begin{matrix} var & covar \\ covar & var \end{matrix} \right|$=$\left| \begin{matrix} a^{2}+c^{2}+e^{2} & {0.25a}^{2}+c^{2} \\ {0.25a}^{2}+c^{2} & a^{2}+c^{2}+e^{2} \end{matrix} \right|$

$a^{2}$ is the estimate of heritability.

All analyses were performed adjusting for sex, birthyear, and birthyear-squared. We fitted models using maximum likelihood and presented standard errors and 95% Wald-type confidence intervals using the function mxSE(). In each model, the thresholds estimated from the phenotype prevalence were allowed to be different between sibling types.

## **References**

1 Ritchie H. 12 key metrics to understand the state of the world - Our World in Data. 2019. https://ourworldindata.org/12-key-metrics (accessed July 15, 2022).

2 GBD 2019 Mental Disorders Collaborators. Global, regional, and national burden of 12 mental disorders in 204 countries and territories, 1990–2019: a systematic analysis for the Global Burden of Disease Study 2019. *Lancet Psychiatry* 2022; **9**: 137–50.

3 Lozano R, Fullman N, Abate D, *et al.* Measuring progress from 1990 to 2017 and projecting attainment to 2030 of the health-related Sustainable Development Goals for 195 countries and territories: a systematic analysis for the Global Burden of Disease Study 2017. *The Lancet* 2018; **392**: 2091–138.

4 Magnus P, Birke C, Vejrup K, *et al.* Cohort Profile Update: The Norwegian Mother and Child Cohort Study (MoBa). *Int J Epidemiol* 2016; **45**: 382–8.

5 WHO Collaborating Centre for Drug Statistics Methodology. Guidelines for ATC classification and DDD assignment. Oslo, 2021.

6 Fazel S, Zetterqvist J, Larsson H, Långström N, Lichtenstein P. Antipsychotics, mood stabilisers, and risk of violent crime. *The Lancet* 2014; **384**: 1206–14.

7 Lagerberg T, Fazel S, Molero Y, *et al.* Associations between selective serotonin reuptake inhibitors and violent crime in adolescents, young, and older adults – a Swedish register-based study. *European Neuropsychopharmacology* 2020; **36**: 1–9.

8 Swedish Board of Health and Welfare. National Guidelines for Treatment of Depression and Anxiety Disorders. 2017.

9 Wray NR, Gottesman II. Using Summary Data from the Danish National Registers to Estimate Heritabilities for Schizophrenia, Bipolar Disorder, and Major Depressive Disorder. *Front Genet* 2012; **3**. DOI:10.3389/FGENE.2012.00118.

10 Boker S, Neale M, Maes H, *et al.* OpenMx: An Open Source Extended Structural Equation Modeling Framework. *Psychometrika* 2011; **76**: 306–17.

11 Falconer DS. The inheritance of liability to certain diseases, estimated from the incidence among relatives. *Ann Hum Genet* 1965; **29**: 51–76.
