# Supplementary material for: Epidemiological overview of major depressive disorder in Scandinavia using nationwide registers
Source: Lancet Reg Health Eur. 2023 Mar 28;29:100621. doi: 10.1016/j.lanepe.2023.100621 (PMC10230616; doi:10.1016/j.lanepe.2023.100621)
Supplement: Supplementary Figs. S1–S12 [file mmc3.docx]

Supplementary Figures

[Figure S0. Flow diagram of the search strategy for the literature reviewed in research in context. 2](#_Toc115789080)

[Figure S1. The dimensional reduction results as a UMAP plot based on the analysis of socioeconomic features. 3](#_Toc115789081)

[Figure S2. The dimensional reduction results as a UMAP plot based on the analysis of morbidity and mortality. 4](#_Toc115789082)

[Figure S3. Cumulative incidence of MDD in Denmark, Norway (MoBa), and Sweden. 5](#_Toc115789083)

[Figure S4. Density plots of age at first specialist diagnosis of MDD in Denmark and Sweden. 6](#_Toc115789084)

[Figure S5. The prevalence of mental health diagnostic chapters in the general population and MDD cases. 9](#_Toc115789085)

[Figure S5. The prevalence of mental health diagnoses (sub codes) in the general population and MDD cases. 12](#_Toc115789086)

[Figure S7. Socioeconomic outcomes in MDD-cases in specialist care versus non-cases. 13](#_Toc115789087)

[Figure S8. Number of treatment contacts in Sweden and Denmark. 14](#_Toc115789088)

[Figure S9. Upset (‘piano’) plot of observed combinations of medications from different groups in MDD cases. 15](#_Toc115789089)

[Figure S10. Yearly country-level use of antidepressants for mood disturbance in Norway. 16](#_Toc115789090)

**Identification of studies via databases and registers**

Records removed *before screening*:

Duplicate records removed (n = 182)

Records marked as ineligible by automation tools (n = 0)

Records removed for other reasons (n =0 )

Records identified from

MEDLINE (n = 141)

Embase (n = 170)

APA PsycInfo (n = 230)

**Identification**

Records screened

(n = 359)

Records excluded*

(n = 0)

Reports sought for retrieval

(n = 359)

Reports not retrieved

(n = 0)

**Screening**

Reports excluded:

Reason 1 (n = 35) Not original article

Reason 2 (n = 61) Did not investigate the whole nation

Reason 3 (n = 185) Investigated MDD as an outcome of other disorders

Reports assessed for eligibility

(n = 359)

Studies included in review

(n = 78)

**Included**

*All articles were screened by one individual for title and abstracts to assess for eligibility.

# Figure S0. Flow diagram of the search strategy for the literature reviewed in research in context.

The database search strategy is summarized in Table S0a and a full list of the reviewed literature can be found in Table S0b.

*From:*  Page MJ, McKenzie JE, Bossuyt PM, Boutron I, Hoffmann TC, Mulrow CD, et al. The PRISMA 2020 statement: an updated guideline for reporting systematic reviews. BMJ 2021;372:n71. doi: 10.1136/bmj.n71


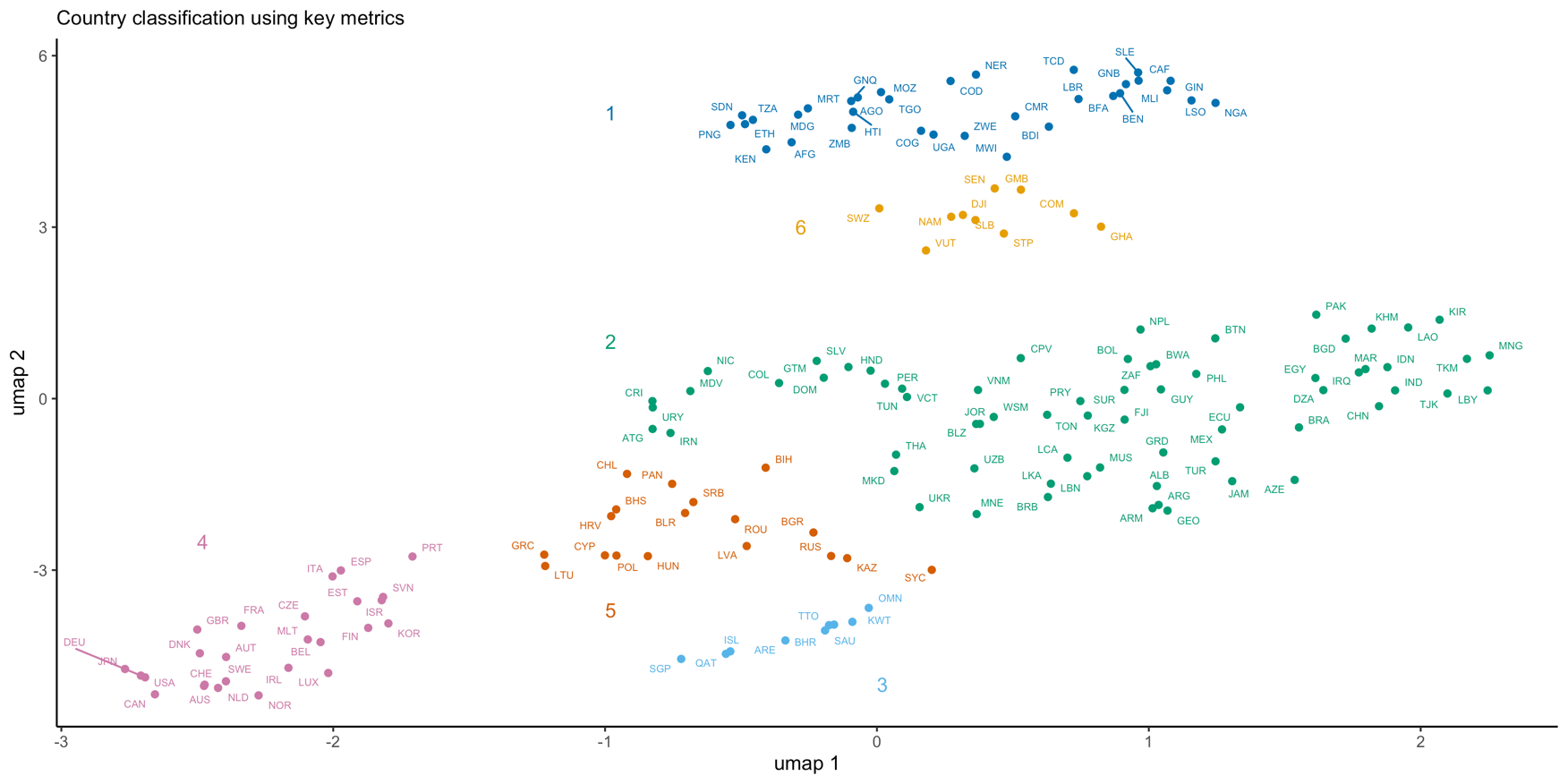


# Figure S1. The dimensional reduction results as a UMAP plot based on the analysis of socioeconomic features.

Features are based on key metrics from Our World in Data. Cluster 4 (pink) is comprised of countries with the most advantageous key metric variables (Table below): most Nordics (Denmark, Estonia, Finland, Norway, Sweden), western Europe (Austria, Belgium, Czechia, France, Germany, Ireland, Italy, Luxembourg, Malta, Netherlands, Portugal, Slovenia, Spain, Switzerland, UK), a few Pacific nations (Australia, Japan, South Korea), North America (Canada, United States), and Israel.

**
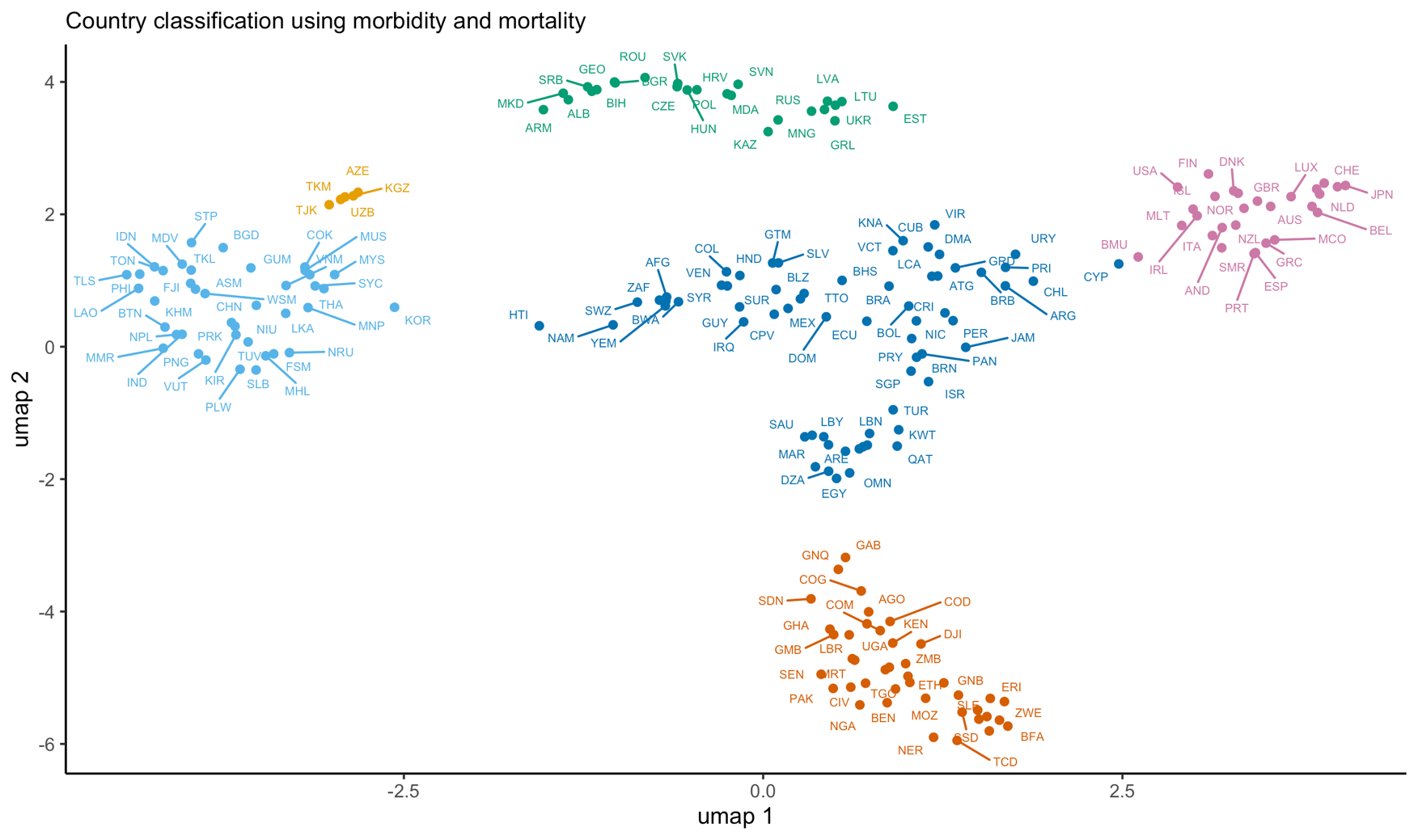
**

# Figure S2. The dimensional reduction results as a UMAP plot based on the analysis of morbidity and mortality.

Coloring is arbitrary and unrelated to key metric graphs. Cluster 4 contains most Nordics (Denmark, Finland, Iceland, Norway, Sweden), much of Europe (Andorra, Austria, Belgium, Cyprus, France, Germany, Greece, Ireland, Italy, Luxembourg, Malta, Monaco, Netherlands, Portugal, San Marino, Spain, Switzerland, UK), a few Pacific nations (Australia, Japan, New Zealand), and North America (Bermuda, Canada, United States).


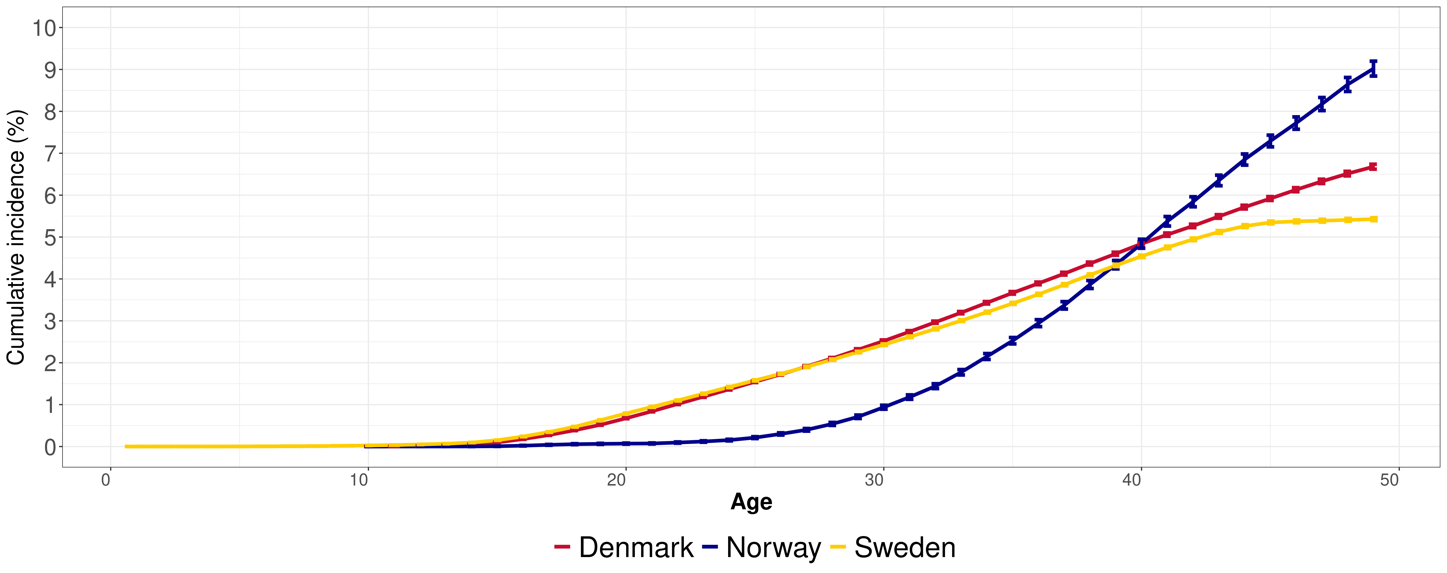


# Figure S3. Cumulative incidence of MDD in Denmark, Norway (MoBa), and Sweden.


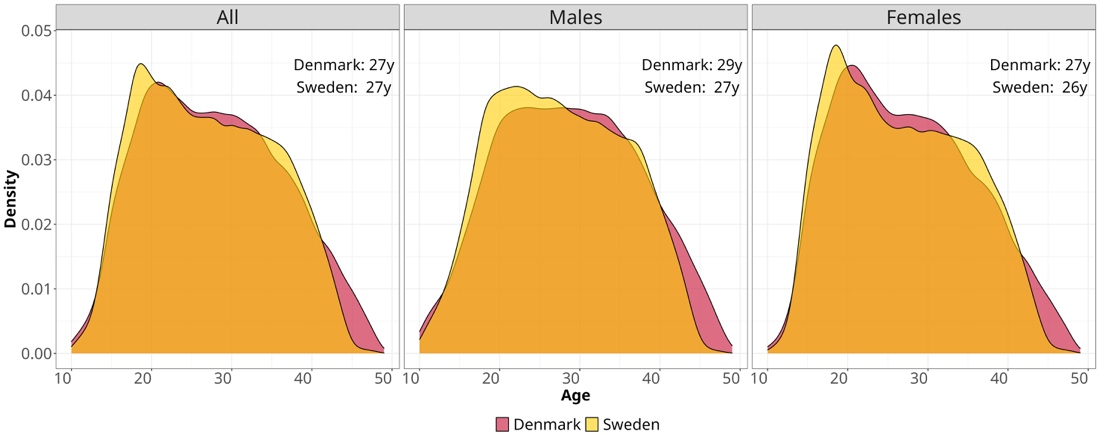


# Figure S4. Density plots of age at first specialist diagnosis of MDD in Denmark and Sweden.

Split out for males and females. Median age at first diagnosis in years (y) is reported in the top corners.


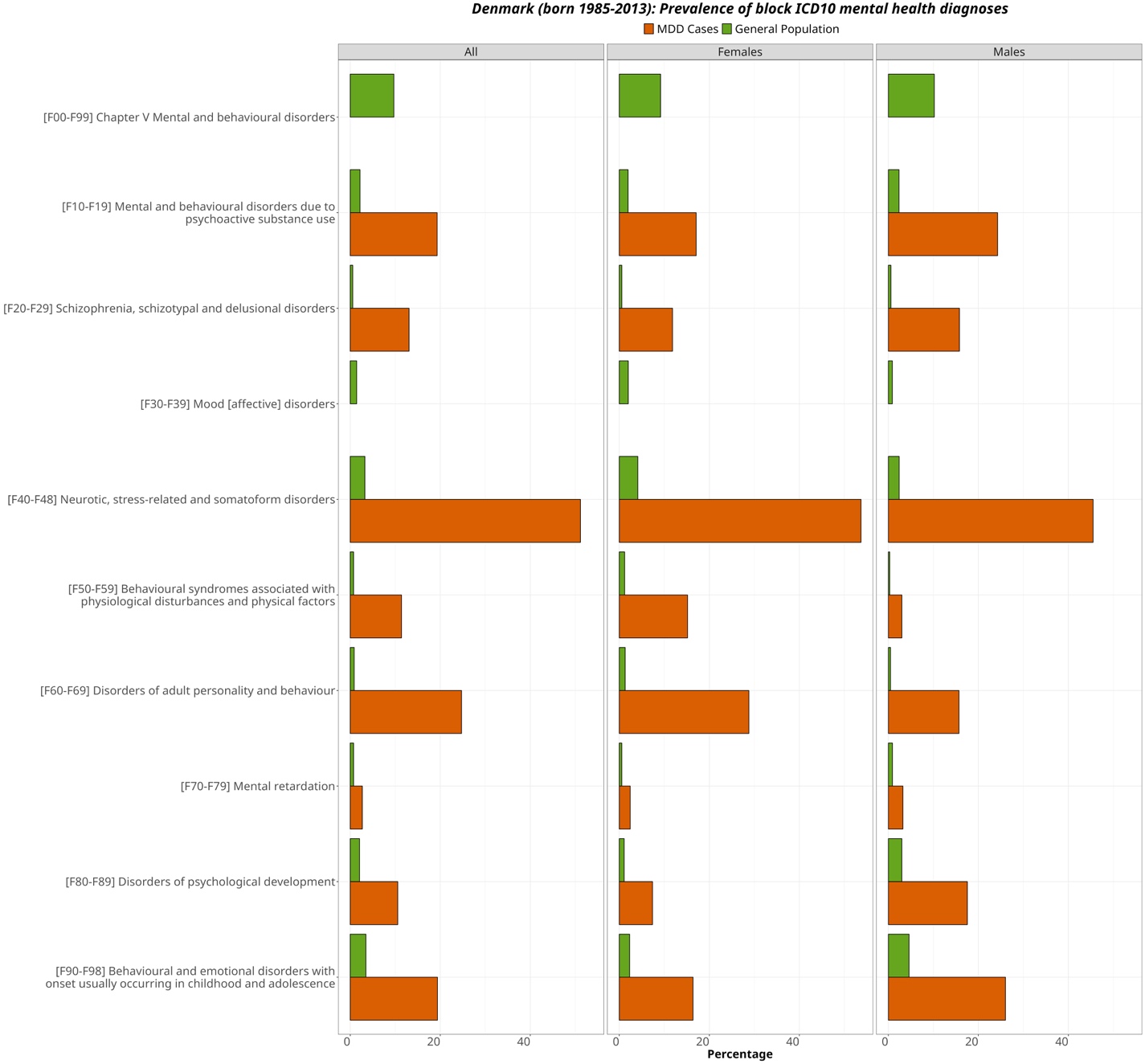


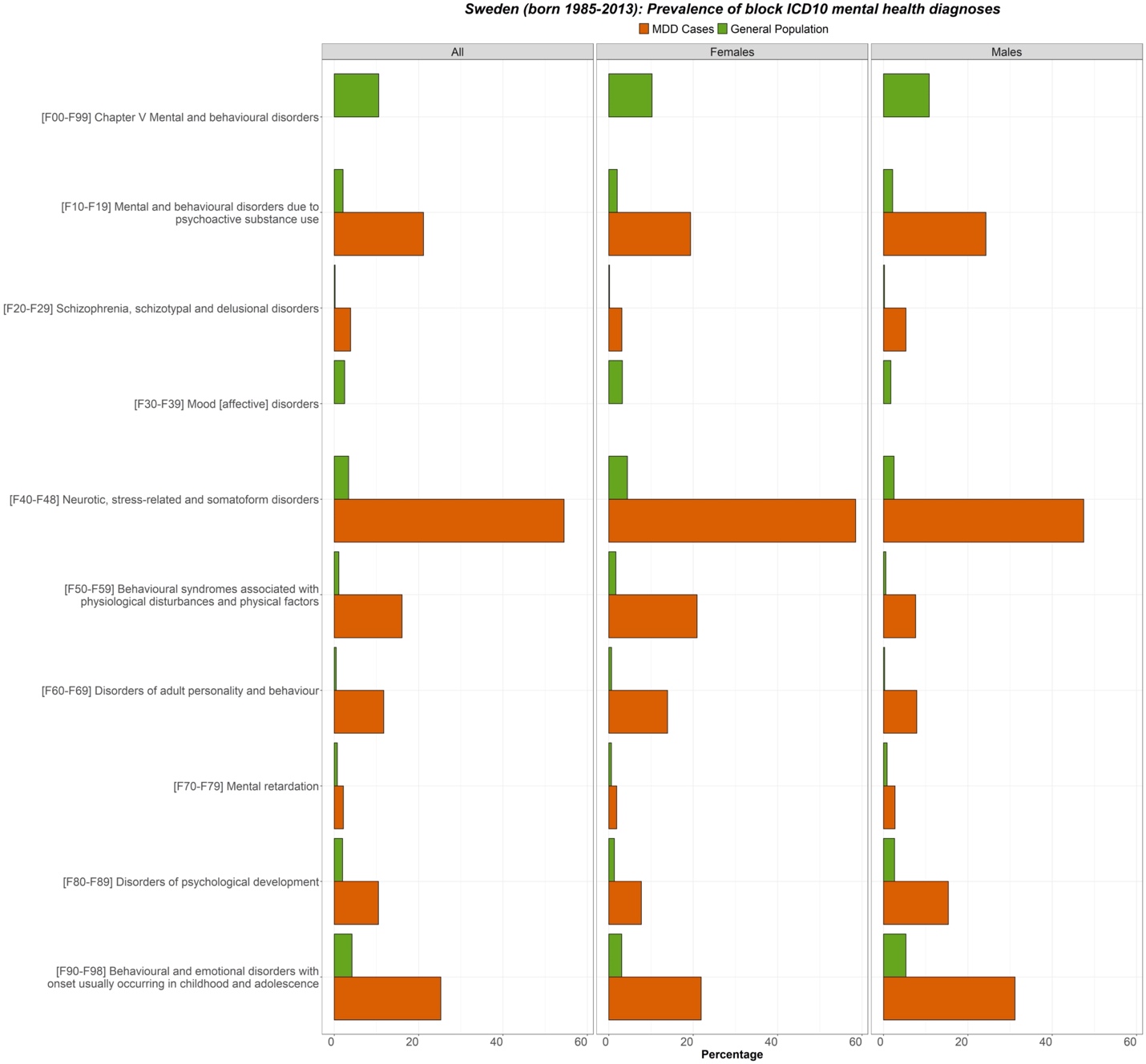


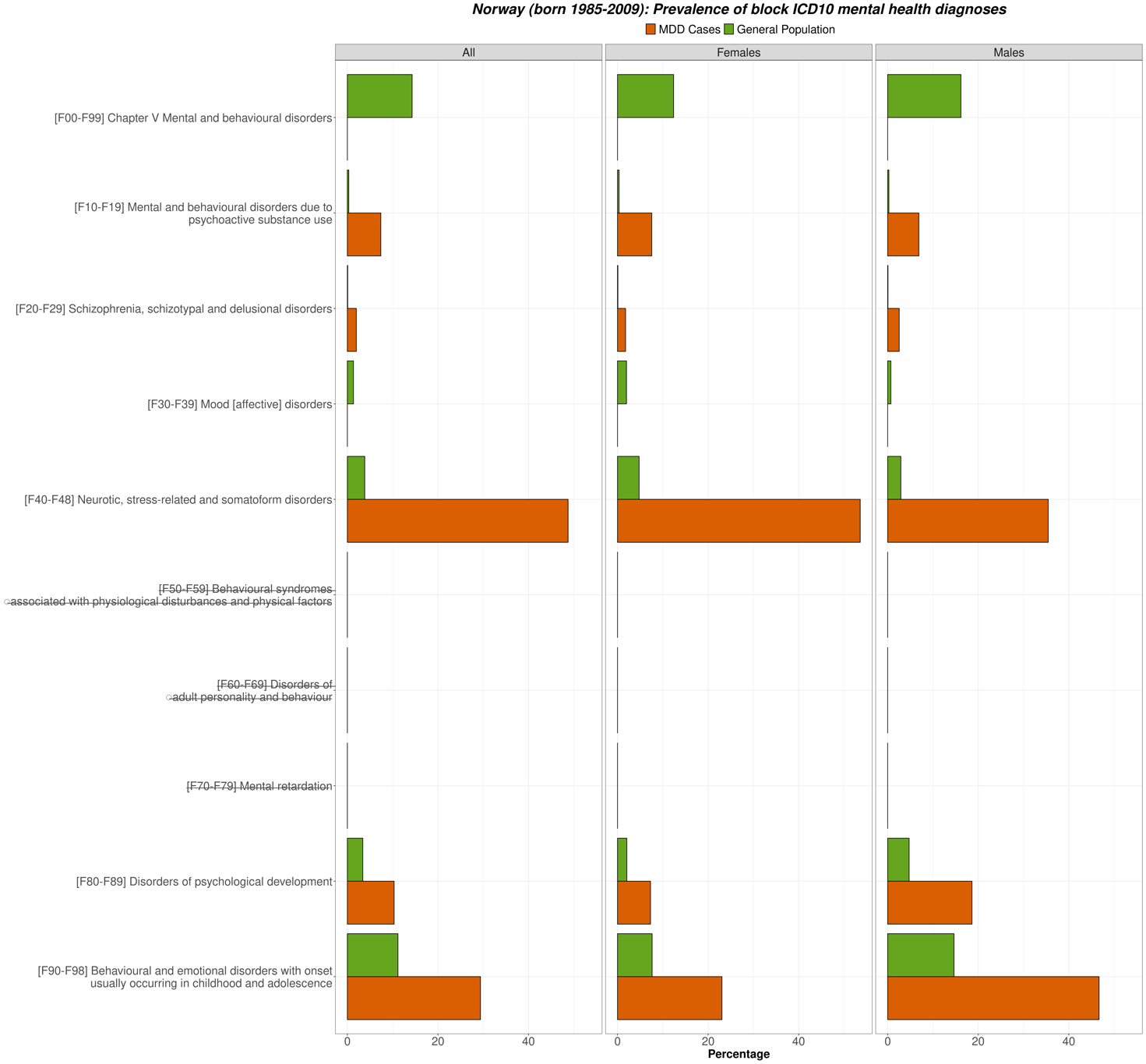


# Figure S5. The prevalence of mental health diagnostic chapters in the general population and MDD cases.

Rates are given per F-chapter in ICD-10 in the general population and in MDD cases in Denmark (top panel), Sweden (center panel), and Norway (MoBa; bottom panel). Percentage is defined as the proportion of cases with at least one registration of the diagnosis in the national specialist health care registries. For reasons of visualization, the percentage of [F00-F99] Chapter V Mental and behavioural disorders are not shown for MDD cases. We excluded F01-F09 disorder, which covers dementia and other mental disorders, due the young birth cohort and late onset of F01-F09 disorder. Chapters that were not available or had a count of less than 6 individuals are shown in strikethrough text.


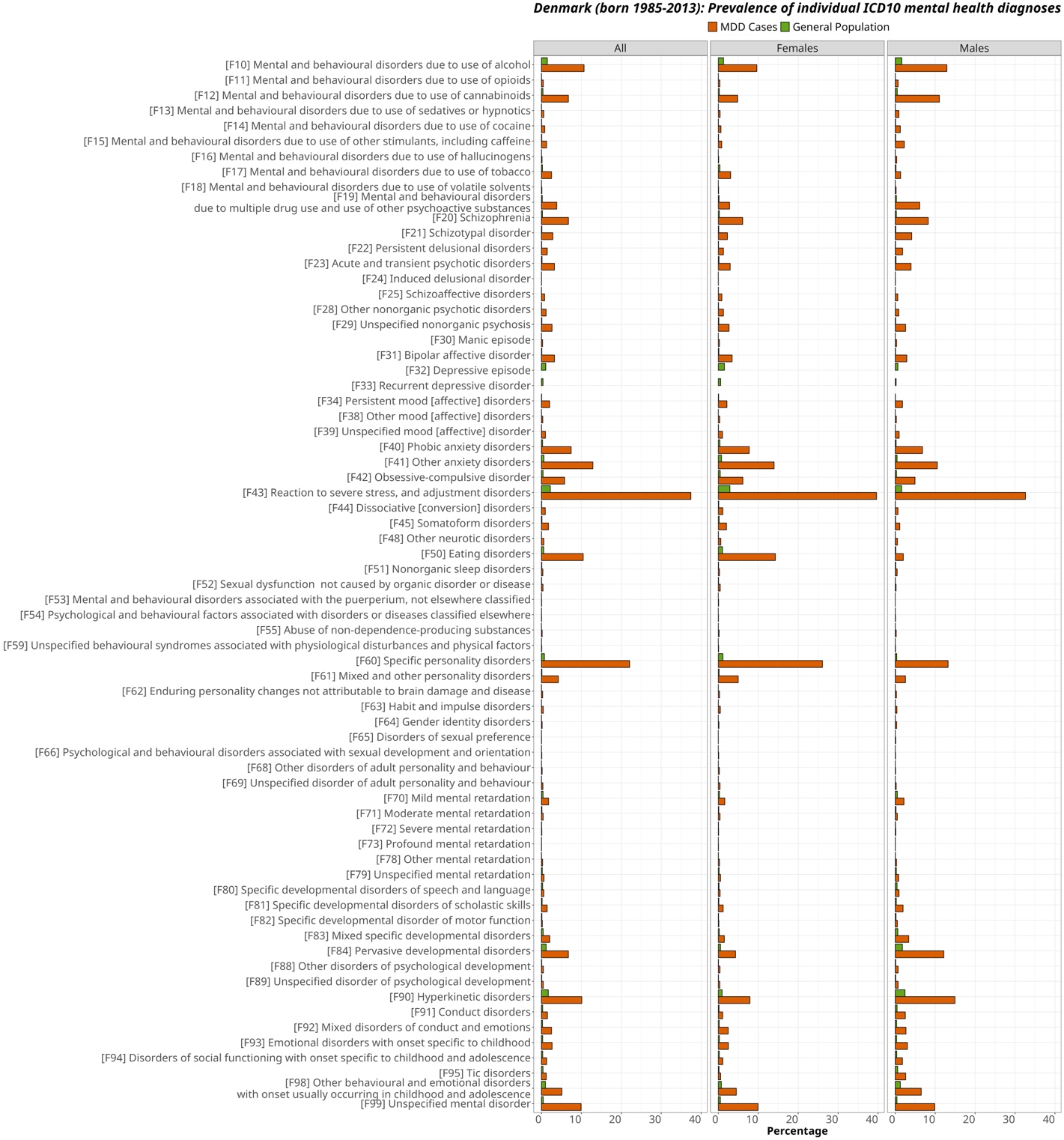


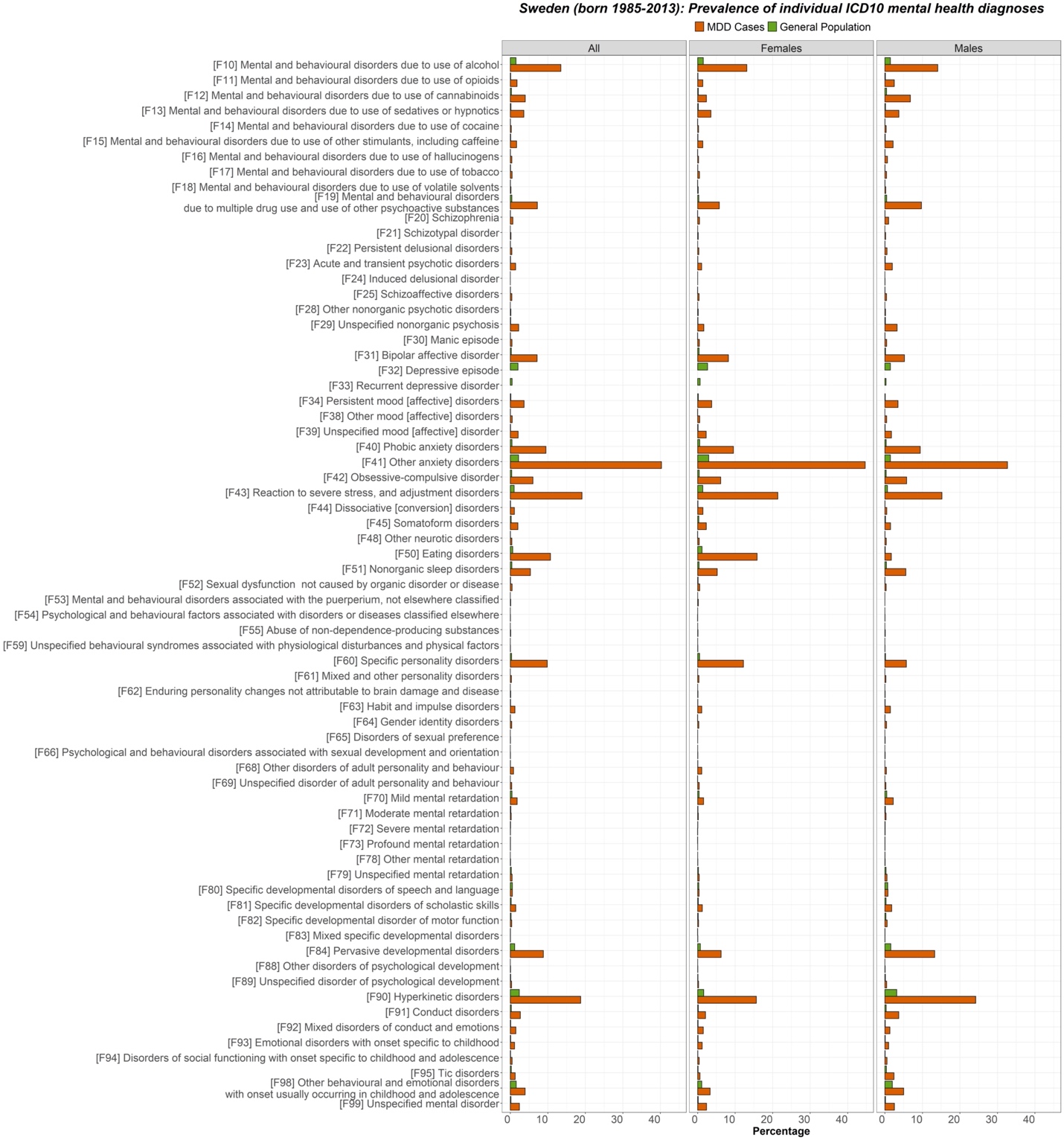


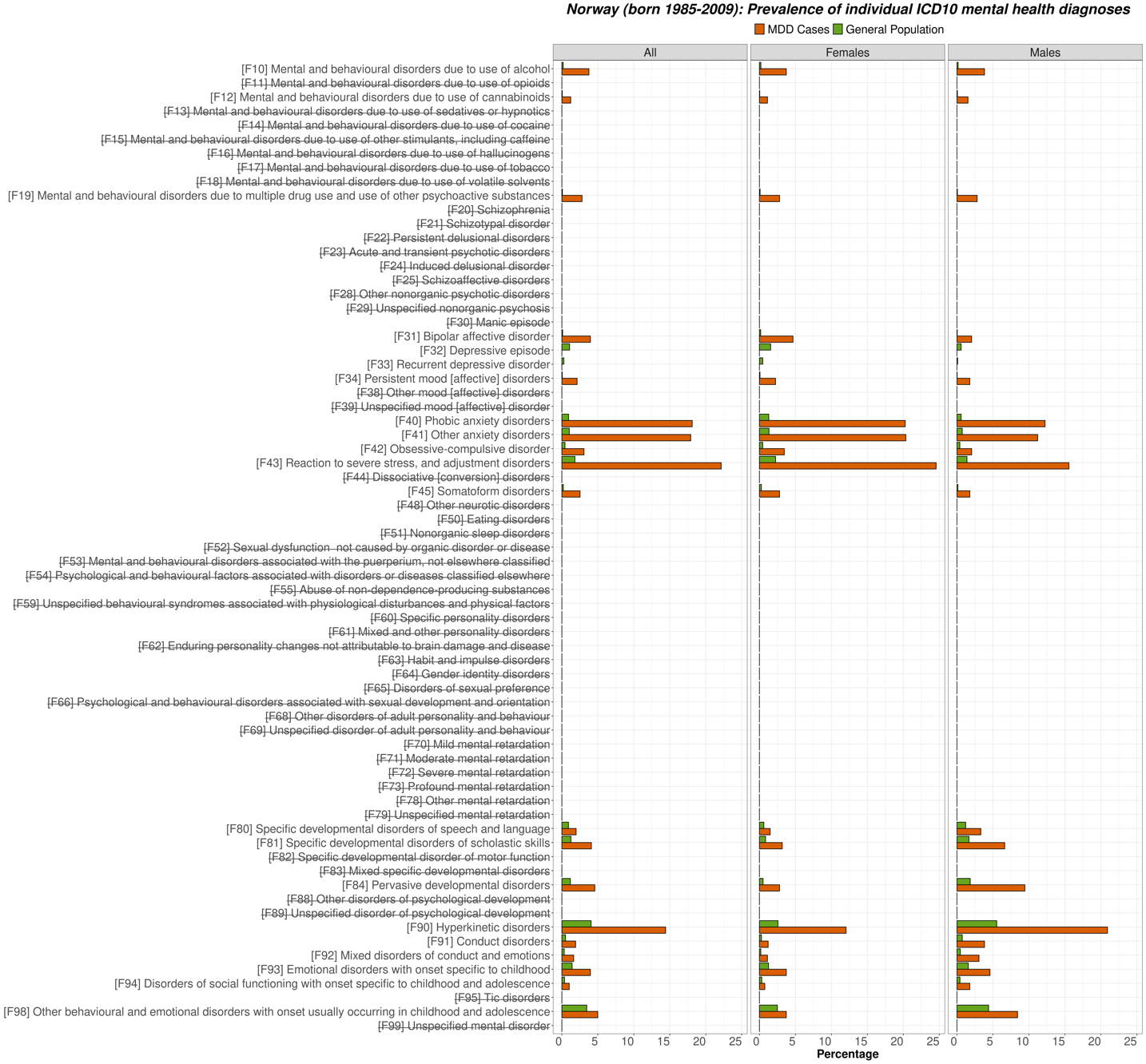


# Figure S6. The prevalence of mental health diagnoses (sub codes) in the general population and MDD cases.

Rates for individual F-codes in ICD-10 are given in the general population and in MDD cases in Denmark (top panel), Sweden (center panel), and Norway (MoBa; bottom panel). Percentage is defined as the proportion of cases with at least one registration of the diagnosis in the national specialist health care registries. For reasons of visualization, the percentage of [F00-F99] Chapter V Mental and behavioural disorders, [F32] Depressive episode, and [F33] Recurrent depressive disorder are not shown for MDD cases. In the Norwegian MoBa sample, F-codes not available for analyses are presented in strikethrough text. We excluded F01-F09 disorder, which covers dementia and other mental disorders, due the young birth cohort and late onset of F01-F09 disorder. Chapters that were not available or had a count of less than 6 individuals are shown in strikethrough text.


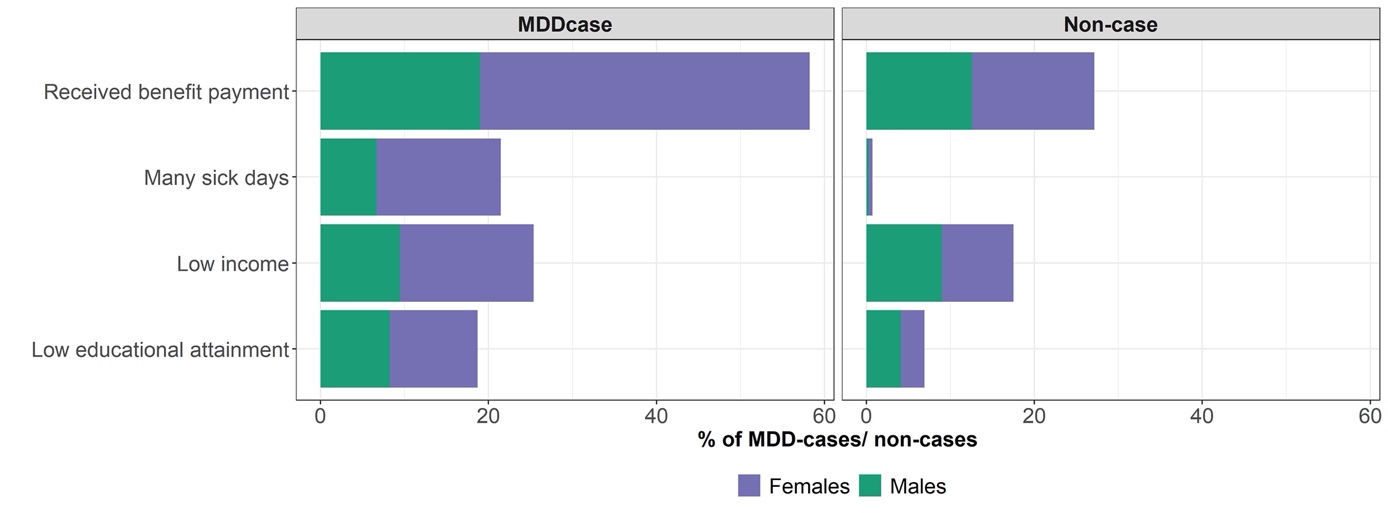


# Figure S7. Socioeconomic outcomes in MDD-cases in specialist care versus non-cases.

Outcomes were measured between 1993-2013. The bars represent the proportion of the population having a high number of sick days (>1SD above average) in the year after diagnosis (for MDD cases) or in any random year (for non-cases), receiving benefit payment for illness or unemployment in any year, having a low level of educational attainment (<1SD below average), or having a low gross monthly income (<1SD below average), stratified by MDD case status and sex.

**
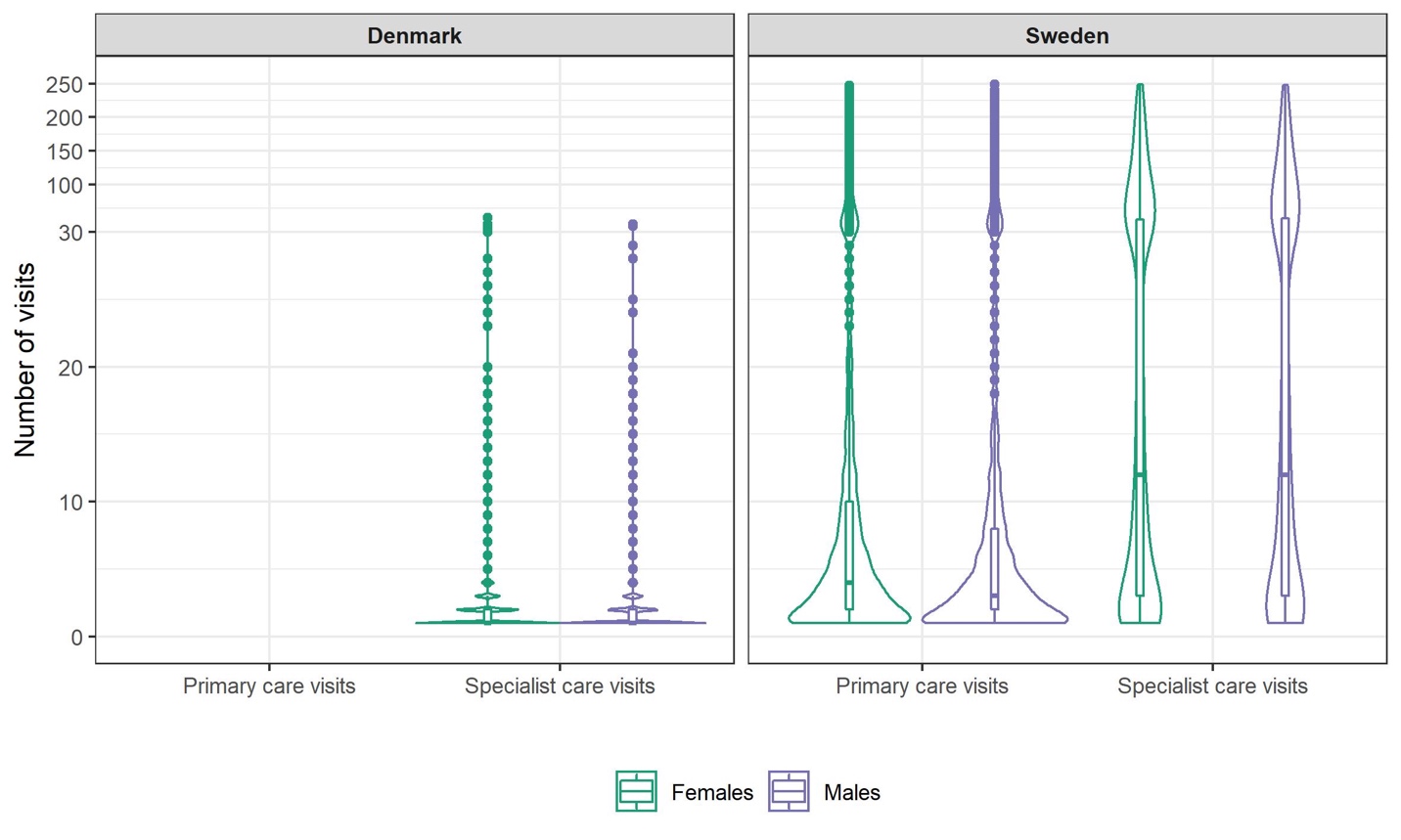
**

# Figure S8. Number of treatment contacts in Sweden and Denmark.

The right part of the panels shows treatment use as the number of visits in specialist care with MDD as the main diagnosis in Denmark (left) and Sweden (right) split out for males and females. In Sweden the number of visits in primary care is also shown. We excluded individuals with more than 250 visits in Sweden (N=2,504).


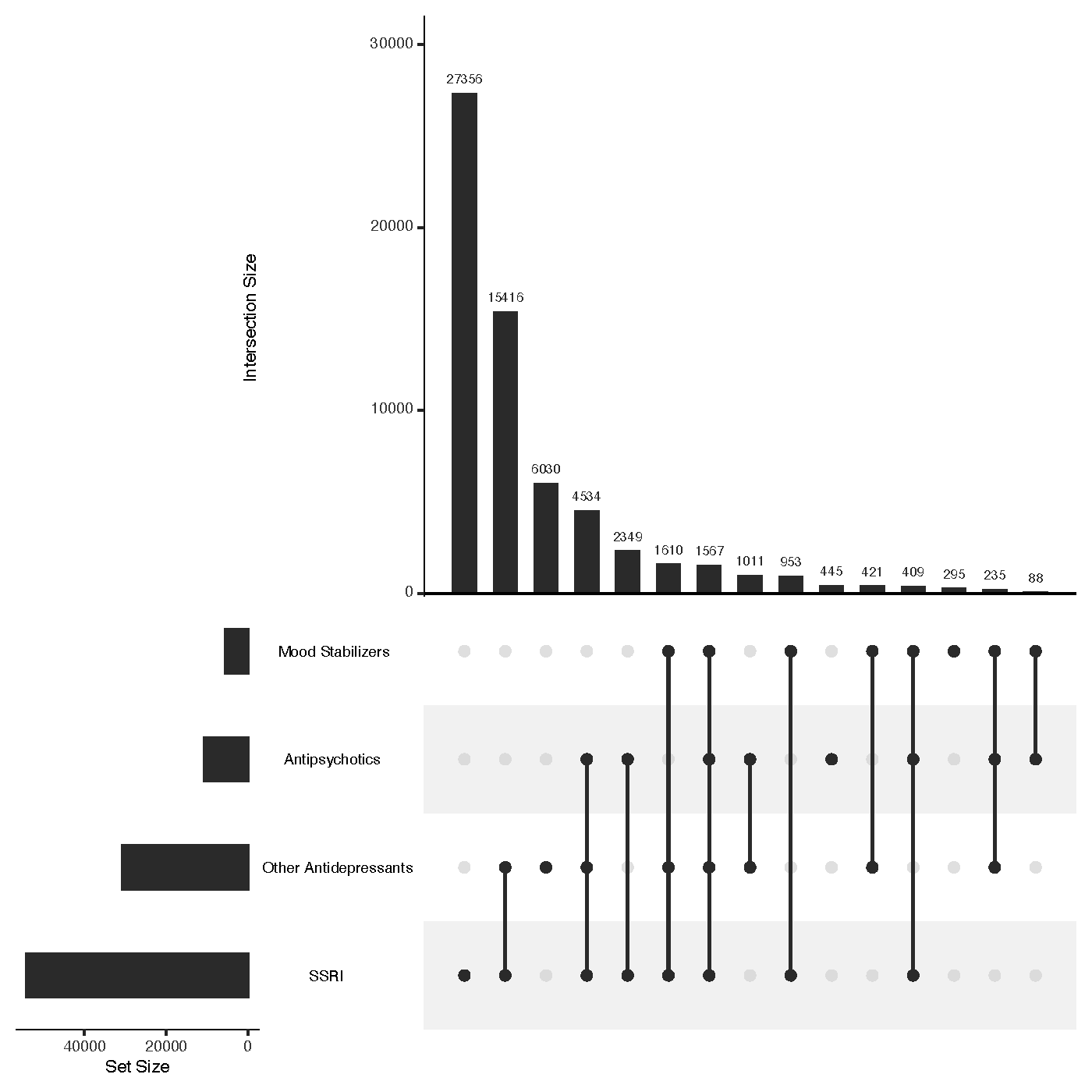


# Figure S9. Upset (‘piano’) plot of observed combinations of medications from different groups in MDD cases.

The right bottom part specifies the possible combinations (e.g., in the second column, having received an SSRI as well as some other antidepressant prescription), with the bars in the top part representing the number of times this pattern is observed in the prescription register (e.g., for SSRI-other antidepressant N=15,416). The set size panel on the left shows the number of prescriptions per medication group.


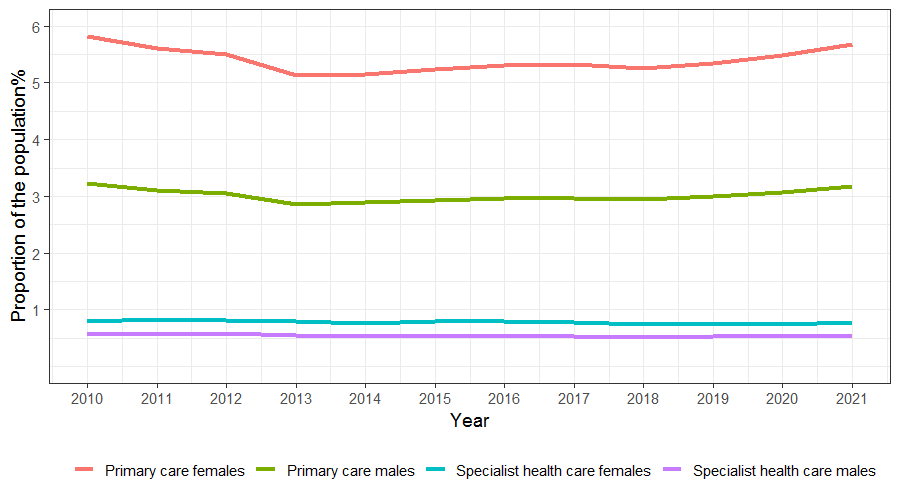


# Figure S10. Yearly country-level use of antidepressants for mood disturbance in Norway.

Data are shown for individuals between 18 and 64 years old.


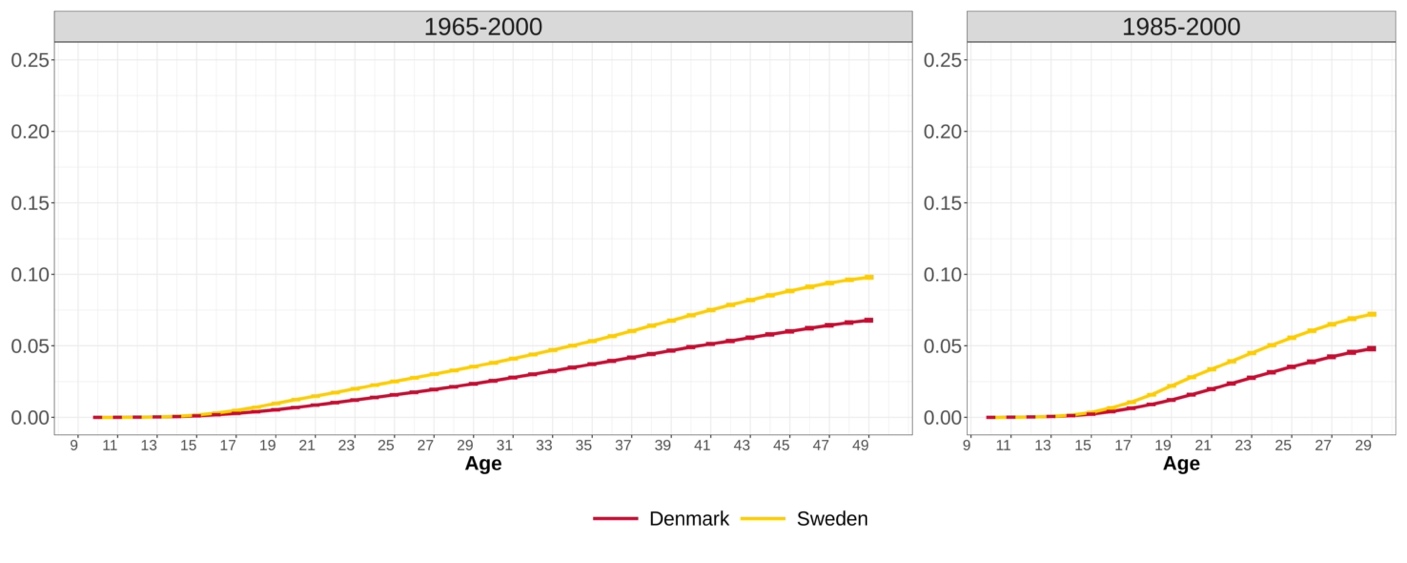


**Figure S11.** Cumulative incidence of MDD in the general population split out by birth cohort and country.


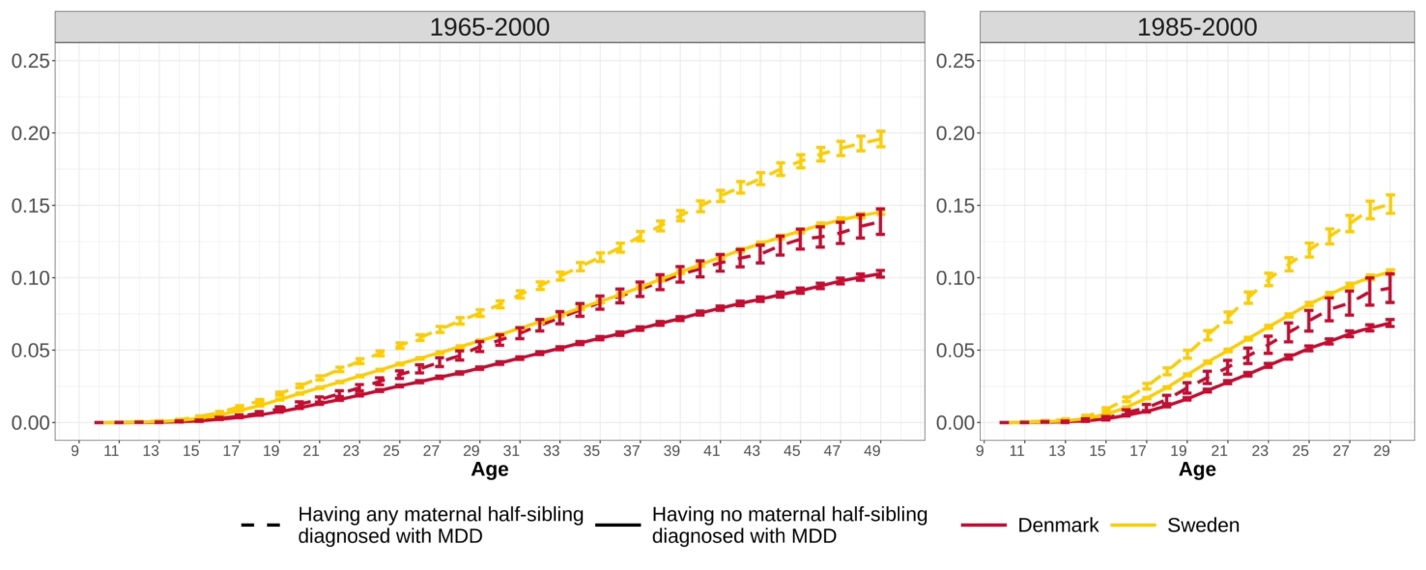


**Figure S12.** Cumulative incidence of MDD as a function of having a maternal half-sibling with MDD, split out by birth cohort and country.
